# Novel genetic variants of *HLA* gene associated with Thai Behcet’s disease (BD) patients using next generation sequencing technology

**DOI:** 10.1038/s41598-024-58254-w

**Published:** 2024-04-04

**Authors:** Gaidganok Sornsamdang, John Shobana, Kumutnart Chanprapaph, Wasun Chantratita, Sasithorn Chotewutmontri, Preeyachat Limtong, Pichaya O-charoen, Chonlaphat Sukasem

**Affiliations:** 1 Division of Pharmacogenomics and Personalized Medicine, Department of Pathology, Faculty of Medicine Ramathibodi Hospital, Mahidol University, Bangkok, 10400 Thailand; 2 Laboratory for Pharmacogenomics, Somdech Phra Debaratana Medical Center (SDMC), Ramathibodi Hospital, Bangkok, Thailand; 3 Pharmacogenomics and Precision Medicine, The Preventive Genomics & Family Check-Up Services Center, Bumrungrad International Hospital, Bangkok, Thailand; 4 Division of Dermatology, Department of Medicine, Faculty of Medicine Ramathibodi Hospital, Mahidol University, Bangkok, Thailand; 5 Center for Medical Genomics, Faculty of Medicine Ramathibodi Hospital, Mahidol University, Bangkok, Thailand; 6 Faculty of Medicine and Public Health, HRH Princess Chulabhorn College of Medical Science, Chulabhorn Royal Academy, Bangkok, Thailand; 7 Division of Allergy, Immunology, and Rheumatology, Department of Medicine, Faculty of Medicine Ramathibodi Hospital, Mahidol University, Bangkok, Thailand; 8 Department of Pharmacology and Therapeutics, MRC Centre for Drug Safety Science, Institute of Systems, Molecular and Integrative Biology, University of Liverpool, Liverpool, UK

**Keywords:** Prognostic markers, Genetic markers

## Abstract

Behçet's disease (BD) manifests as an autoimmune disorder featuring recurrent ulcers and multi-organ involvement, influenced by genetic factors associated with both *HLA* and non-*HLA* genes, including *TNF-α* and *ERAP1*. The study investigated the susceptible alleles of both Class I and II molecules of the *HLA* gene in 56 Thai BD patients and 192 healthy controls through next-generation sequencing using a PacBio kit. The study assessed 56 BD patients, primarily females (58.9%), revealing diverse manifestations including ocular (41.1%), vascular (35.7%), skin (55.4%), CNS (5.4%), and GI system (10.7%) involvement. This study found associations between BD and HLA-A*26:01:01 (OR 3.285, 95% CI 1.135–9.504, *P*-value 0.028), HLA-B*39:01:01 (OR 6.176, 95% CI 1.428–26.712, *P*-value 0.015), HLA-B*51:01:01 (OR 3.033, 95% CI 1.135–8.103, *P*-value 0.027), HLA-B*51:01:02 (OR 6.176, 95% CI 1.428–26.712, *P*-value 0.015), HLA-C*14:02:01 (OR 3.485, 95% CI 1.339–9.065, *P*-value 0.01), HLA-DRB1*14:54:01 (OR 1.924, 95% CI 1.051–3.522, *P*-value 0.034), and HLA-DQB1*05:03:01 (OR 3.00, 95% CI 1.323–6.798, *P*-value 0.008). However, after Bonferroni correction none of these alleles were found to be associated with BD. In haplotype analysis, we found a strong linkage disequilibrium in HLA-B*51:01:01, HLA-C*14:02:01 (*P*-value 0.0, Pc-value 0.02). Regarding the phenotype, a significant association was found between HLA-DRB1*14:54:01 (OR 11.67, 95% CI 2.86–47.57, *P*-value 0.001) and BD with ocular involvement, apart from this, no distinct phenotype-*HLA* association was documented. In summary, our study identifies specific *HLA* associations in BD. Although limited by a small sample size, we acknowledge the need for further investigation into *HLA* relationships with CNS, GI, and neurological phenotypes in the Thai population.

## Introduction

Behçet's disease (BD) is an autoimmune disorder characterized by recurrent ulcers in the mouth, genitalia, and eyes, with potential multi-organ involvement^1,2^. It is classified as variable vessel vasculitis due to its impact on blood vessels^3^. The prevalence of BD is higher in countries along the Silk Road, particularly in the Mediterranean and northern East Asia^4,5^. Epidemiological studies reveal varying rates, such as 420 per 100,000 in Turkey^6^ and 35 per 100,000 in Korea, marking the highest prevalence among Asian nations^7^. In Thailand, a 24-year study (1980–2003) documented 23 cases of BD^8^. Initially believed to be an auto-inflammatory disease, recent studies suggest it is an auto-inflammatory and auto-immune disorder, with innate immune mutations, vascular diseases, and co-morbid conditions contributing to the auto-inflammatory side and adaptive immune system mutations causing the autoimmune disorder^9–11^.

Genetics are the most significant risk factors for BD, with HLA-B*51 allele variations present in 50–60% of patients^12^. The precise role of HLA-B*51 in Behçet's syndrome remains uncertain, with ongoing debates about whether it is directly involved in the disease or merely acts as a marker. Other *HLA* and non-*HLA* alleles have also been linked to BD. For example, the systematic review, encompassing 11 studies, highlights increased TNF-α production, potentially triggered by TLR-signalling, and underscores TNF-α's pivotal role in BD's immunopathogenesis^13^***.*** A study in Spain found no significant association between Behçet's disease (BD) and multiple *ERAP1* polymorphisms, but increased frequencies of these polymorphisms was seen in patients with *HLA-B* risk suggesting a potential epistatic interaction between *ERAP1* and *HLA-B*^14^***.*** Many studies reported this higher likelihood of developing BD in HLA-B*51 positive cases along with homozygosity for the *ERAP1*^15,16^. Additionally, independent risk alleles such as *HLA-B ∗ 15*, *HLA-B ∗ 27*, *HLA-B ∗ 57*, HLA-A ∗ 26, HLA-A*02, HLA-A*27, HLA-B*57, *MICA-TM-A6* and *TNFX103-1C* have been identified in the development of Behçet's syndrome^17,18^.

HLA-B*51 stands out as the most significant genetic factor in Behcet's syndrome, and its prevalence exhibits variations among clinical clusters^19^. A meta-analysis involving 4800 patients and 289 healthy controls suggested a 32–52% attributable risk for HLA-B*51^12^. While numerous *HLA* alleles have been reported as susceptible in various Asian and European countries, limited studies have explored their association with BD in the Southeast Asian (SEA) population. Earlier studies in Thailand identified HLA-B*51:01 as a major susceptible allele in the Thai population, with HLA-A*26:01 reported as a risk allele in non-carriers of HLA-B*51:01^20^. BD's most prevalent clinical manifestations involve the skin (38–99%), mucous membranes (mouth 47–86%, genitals 57–93%, eyes 30–70%)^21,22^, joints (45–60%), cardiovascular system (CVS 9–49%), neurological system (5–10%), and gastrointestinal system (3–26%). Poor prognosis^23^ is associated with BD patients having ocular^24^, cardiovascular, central nervous system (CNS), or gastrointestinal involvement. Risk alleles for specific clinical manifestations or phenotypes may vary; for example, a Korean study identified HLA-A*02:07 as a risk allele for skin lesions and arthritis, and HLA-A*26:01, HLA-A*30:04, and HLA-B*51:01 as risk alleles for uveitis, vasculitis, and BD, respectively^25^.

High-resolution *HLA* typing is instrumental in pinpointing specific *HLA* alleles, offering a more precise understanding of an individual's *HLA* profile and its correlation with BD. Notably, a study in Turkey identified a susceptibility association with HLA-B*51 carriers, while HLA-B*52 was found to have a protective effect^26^. Similarly, an Italian study revealed a high frequency of the HLA-B*51 allele (62.7%), with HLA-B*51:01 and HLA-B*51:08 being the most common subtypes. The occurrence of ocular involvement was statistically linked to HLA-B*51 positivity and the HLA-B*51:01 and HLA-B*51:08 subtypes^27^*.* Another NGS study from Egypt implicated HLA-B*51:08 in legal blindness among BD patients^28^. Overall, high-resolution *HLA* typing offers a more detailed and clinically relevant perspective on an individual's *HLA* alleles, enhancing medical decision-making and enabling personalized treatment strategies for BD. This study in Thailand is among the first to explore the association of both class I and II *HLA* molecules with BD in high resolution (6-digits) using NGS technology.

## Results

### Patients’ demographic data and clinical characteristics

According to the ICBD criteria, 17 (30.4%) of the fifty-six BD patients had 4 scores, 24 (42.3%) had 5 scores, and 15 (26.8%) had 6 scores. Twenty-three (41.1%) patients were males, and 33 (58.9%) were females, with an average age of onset of 35.5 years (range age of onset: 12–54 years). Ocular, vascular, skin, CNS, and GI systems are the most involved systems in BD. All BD patients had oral ulcers (56, 100%), followed by genital ulcers 34 (60.7%), uveitis 19 (33.9%), retinal vasculitis 15 (26.8%), epidermal necrolysis (EN) 20 (35.7%), papulopustular 20 (35.7%), leg ulcers 1 (1.8%), vasculitis 8 (14.3%), and arthritis 17 (30.4%). In this study, 23 (41.1%) patients had ocular involvement, which included uveitis and retinal vasculitis. Twenty patients (or 35.7%) had vascular involvement, including vasculitis (thrombophlebitis and deep vein thrombosis) and retinal vasculitis. Epidermal necrolysis (EN), papulopustular lesions, and leg ulcers were the skin involvements that were observed in 31 patients (55.4%). The CNS and GI systems were involved in 3 (5.36%) and 6 (10.7%) patients, respectively. Ten patients (16.1%) had a positive allergy test. Of the one hundred and ninety-two controls, 111 (57.8%) were male and 81 (42.2%) were female, with an average age of 60.8 years (range 60–74 years). Case and control characteristics and clinical data are summarised in Table 1.

**Table 1:** Demographics and clinical characteristics of the study population.

Demographic data	BD (n = 56)	Control (n = 192)	*P*-value
Sex, n (%)			
Male	23 (41.1)	111 (57.8)	0.027
Female	33 (58.9)	81 (42.2)	–
Age Onset (mean/median)	35.5/35.5	–	–
Age (mean/median)	–	60.8 (59)	–
Age (min/max)	12/54	60/74	–
ICBD criteria (n, %)			
Probable (BD score = 4)	17 (30.4)	–	–
BD Highly likely (BD score = 5)	24 (42.3)	–	–
Almost Certainly (BD score = 6	15 (26.8)	–	–
Clinical data (n, %)			
Oral Ulcer	56 (100)	–	–
Genital Ulcer	34 (60.7)	–	–
Uveitis	19 (33.9)	–	–
Retinal vasculitis	15 (26.8)	–	–
Epidermal Necrolysis (EN)	20 (35.7)	–	–
Papulopustular	20 (35.7)	–	–
Leg Ulcer	1 (1.8)	–	–
Vasculitis	8 (14.3)	–	–
Arthritis	17 (30.4)	–	–
Organ involvement			
Ocular (Uveitis, Retinal Vasculitis)	23 (41.1)	–	–
Vascular (Vasculitis, Retinal vasculitis)	20 (35.7)	–	–
Skin (EN, Papulopustular, leg ulcer)	31 (55.4)	–	–
Central Nervous system (CNS)	3 (5.4)	–	–
Gastro–Intestinal tract (GI)	6 (10.7)	–	–
Pathergy Test, Positive	10 (17.9)	–	–

*BD* Behcet’s Disease, *ICBD* The international for Criteria Bechet’s Disease.

### The association between various *HLA *Class I and *HLA* Class II molecules using next generation sequencing (NGS)

The univariate analysis of this study showed that HLA-A*26:01:01 had an association with BD. This allele was carried by 4.7% of control and 12.5% of case groups respectively (OR-3.285, *P*-value = 0.028, 95% CI = 1.135–9.504). Other *HLA-B* alleles that have been shown to be strongly associated with BD that includes HLA-B*39:01:01 (OR = 6.16, 95% CI = 1.428–26.712, *P*-value = 0.015), HLA-B*51:01:01 (OR = 3.033, 95% CI = 1.135–8.103, P-value = 0.027), and HLA-B*51:01:02 (OR = 6.176, 95% CI = 1.428–26.712, *P*-value = 0.005). In *HLA-C*, HLA-C*14:02:01 was also found to be associated with BD (OR = 3.485, 95% CI = 1.339–9.065, *P*-value = 0.001) (Table 2). The univariate analysis of *HLA* Class II alleles such as HLA-DRB1*14:54:01 (OR 1.924, 95% CI = 1.051–3.522, *P*-value = 0.034) and HLA DQB1*05:03:01 (OR = 3.00, 95% CI = 1.323–6.798, *P*-value = 0.008) were found to be associated with BD as well (Table 3). For the multivariate analysis, P-value were adjusted by using Bonferroni’s correction (34 for *HLA-A*; HLA-A*26:01:01:01 with Pc-value = 0.952, 49 for *HLA-B*; HLA-B*39:01:01 and HLA-B*51:01:02 with Pc-value = 0.735, HLA-B*51:01:01 with Pc-value = 1.323, 24 for *HLA-C*; HLA-C*14:02:01 with Pc-value = 0.240, 27 for *HLA-DRB1*; HLA-DRB1*14:54:01 with Pc-value-0.918, and 16 for *HLA-DQB1*; HLA-DQB1*05:03:01 with Pc-value = 0.128), there have no *HLA* alleles shown associated with BD.

**Table 2:** Analysis of various *HLA* Class I molecules and BD associations using NGS.

*HLA-A* 6-Digit	Control BD n = 192, n (%)	BD n = 56, n (%)	Odds Ratio	*P*-value	95%CI	*HLA-B* 6-Digit	Control BD n = 192, n (%)	BD n = 56, n (%)	Odds Ratio	*P*-value	95%CI
A*01:01:01	10 (5.21)	1 (1.79)	0.330	0.297	0.041–2.642	B*07:02:01	12 (6.25)	2 (3.57)	0.555	0.451	0.120–2.559
A*02:01:01	12 (6.25)	3 (5.36)	0.849	0.805	0.2310–3.120	B*07:05:01	2 (1.04)	0 (0.00)	–	–	–
A*02:01:13	1 (0.52)	0 (0.00)	–	–	–	B*08:01:01	1 (0.52)	0 (0.00)	–	–	–
A*02:03:01	31 (16.15)	9 (16.07)	0.995	0.989	0.442–2.235	B*13:01:01	15 (7.81)	6 (10.71)	1.416	0.494	0.522–3.839
A*02:06:01	5 (2.60)	0 (0.00)	–	–	–	B*13:02:01	9 (4.69)	4 (7.14)	1.564	0.471	0.462–5.284
A*02:07:01	35 (18.23)	13 (23.21)	1.356	0.407	0.659–2.787	B*15:01:01	7 (3.65)	1 (1.79)	0.480	0.497	0.057–3.990
A*02:09:01	1 (0.52)	1 (1.79)	3.472	0.381	0.213–56.425	B*15:02:01	33 (17.19)	7 (12.50)	0.688	0.403	0.286–1.653
A*02:11:01	1 (0.52)	1 (1.79)	3.472	0.381	0.213–56.425	B*15:07:01	0 (0.00)	2 (3.57)	–	–	–
A*02:17:02	1 (0.52)	0 (0.00)	–	–	–	B*15:11:01	2 (1.04)	0 (0.00)	–	–	–
A*03:01:01	9 (4.69)	0 (0.00)	–	–	–	B*15:12:02	4 (2.08)	0 (0.00)	–	–	–
A*03:08:01	0 (0.00)	1 (1.79)	–	–	–	B*15:15:01	1 (0.52)	0 (0.00)	–	–	–
A*11:01:01	82 (42.71)	22 (39.29)	0.868	0.648	0.472–1.593	B*15:18:01	3 (1.56)	0 (0.00)	–	–	–
A*11:01:75	0 (0.00)	1 (1.79)	–	–	–	B*15:21:01	1 (0.52)	0 (0.00)	–	–	–
A*11:01:82	1 (0.52)	0 (0.00)	–	–	–	B*15:25:01	4 (2.08)	2 (3.57)	1.740	0.529	0.310–9.761
A*11:02:01	8 (4.17)	0 (0.00)	–	–	–	B*15:32:01	2 (1.04)	0 (0.00)	–	–	
A*23:01:01	1 (0.52)	0 (0.00)	–	–	–	B*18:01:01	16 (8.33)	6 (10.71)	1.320	0.582	0.490–3.550
A*24:02:01	43 (22.40)	16 (28.57)	1.386	0.341	0.708–2.713	B*27:04:01	14 (7.29)	3 (5.36)	0.719	0.616	0.199–2.599
A*24:02:40	3 (1.56)	0 (0.00)	–	–	–	B*27:05:02	2 (1.04)	0 (0.00)	–	–	–
A*24:03:01	4 (2.08)	1 (1.79)	0.854	0.889	0.093–7.803	B*35:01:01	5 (2.60)	0 (0.00)	–	–	–
A*24:07:01	13 (6.77)	3 (5.36)	0.779	0.705	0.214–2.837	B*35:03:01	4 (2.08)	0 (0.00)	–	–	–
A*24:10:01	7 (3.65)	4 (7.14)	2.032	0.272	0.572–7.213	B*35:05:01	8 (4.17)	1 (1.79)	0.418	0.416	0.051–3.416
A*24:20:01	4 (2.08)	1 (1.79)	0.854	0.889	0.093–7.803	B*37:01:01	7 (3.65)	0 (0.00)	–	–	
A*26:01:01	**8 (4.17)**	**7 (12.50)**	***3.285***	***0.028***	***1.135–9.504***	B*38:02:01	13 (6.77)	1 (1.79)	0.250	0.187	0.032–1.956
A*29:01:01	2 (1.04)	1 (1.79)	1.727	0.658	0.153–19.408	B*39:01:01	**3 (1.56)**	**5 (8.93)**	**6.176**	**0.015**	***1.428–26.712***
A*30:01:01	10 (5.21)	4 (7.14)	1.400	0.583	0.421–4.647	B*39:09:01	2 (1.04)	2 (3.57)	3.518	0.214	0.484–25.563
A*30:01:12	1 (0.52)	0 (0.00)	–	–	–	B*40:01:02	31 (16.15)	9 (16.07)	0.994	0.989	0.442–2.235
A*31:01:02	4 (2.08)	1 (1.79)	0.854	0.889	0.093–1.803	B*40:02:01	9 (4.69)	2 (3.57)	0.753	0.722	0.157–3.590
A*32:01:01	3 (1.56)	1 (1.79)	1.145	0.907	0.116–11.232	B*40:06:01	7 (3.65)	1 (1.79)	0.480	0.497	0.057–3.990
A*33:01:01	1 (0.52)	0 (0.00)	–	–	–	B*44:02:01	0 (0.00)	2 (3.57)	–	–	–
A*33:03:01	49 (25.52)	15 (26.79)	1.067	0.849	0.543–2.096	B*44:03:01	0 (0.00)	1 (1.79)	–	–	–
A*34:01:01	1 (0.52)	0 (0.00)	–	–	–	B*44:03:02	24 (12.50)	10 (17.86)	1.521	0.308	0.679–3.409
A*68:01:01	2 (1.04)	0 (0.00)	–	–	–	B*46:01:01	46 (23.96)	14 (25.00)	1.057	0.873	0.530–2.108
A*68:01:02	1 (0.52)	0 (0.00)	–	–	–	B*46:01:19	0 (0.00)	1 (1.79)	–	–	–
A*74:02:01	5 (2.60)	1 (1.79)	0.679	0.727	0.0777–5.943	B*48:01:01	5 (2.60)	0 (0.00)	–	–	–
						B*50:01:01	0 (0.00)	1 (1.79)	–	–	–
						B*51:01:01	**10 (5.21)**	**8 (14.29)**	**3.033**	**0.027**	***1.135–8.103***
						B*51:01:02	**3 (1.56)**	**5 (8.93)**	**6.176**	**0.015**	***1.428–26.712***
						B*51:02:01	5 (2.60)	0 (0.00)	–	–	–
						B*51:02:02	1 (0.52)	1 (1.79)	3.472	0.381	0.213–56.425
						B*51:06:01	1 (0.52)	0 (0.00)	–	–	–
						B*52:01:01	10 (5.21)	2 (3.57)	0.674	0.618	0.1431–3.170
						B*53:01:01	1 (0.52)	0 (0.00)	–	–	–
						B*54:01:01	6 (3.13)	1 (1.79)	0.563	0.599	0.066–4.782
						B*55:01:01	5 (2.60)	1 (1.79)	0.679	0.727	0.077–5.943
						B*55:02:01	2 (1.04)	0 (0.00)	–	–	–
						B*56:01:01	4 (2.08)	2 (3.57)	1.740	0.529	0.310–9.761
						B*57:01:01	6 (3.13)	2 (3.57)	1.148	0.868	0.225–5.852
						B*58:01:01	22 (11.46)	4 (7.14)	0.594	0.358	0.195–1.803
						B*67:01:01	1 (0.52)	0 (0.00)	–	–	–

*HLA-C* 6-Digit	Control n = 192, n (%)	BD n = 56, n (%)	Odds Ratio	*P*-value	95%CI						
C*01:02:01	54	19	1.312	0.403	0.694–2.479						
C*02:02:02	2	0	–	–	–						
C*03:02:02	23	4	0.565	0.312	0.186–1.708						
C*03:03:01	12	2	0.555	0.451	0.120–2.559						
C*03:04:01	25	11	1.632	0.219	0.747–3.568						
C*04:01:01	21	1	0.148	0.065	0.019–1.126						
C*04:03:01	8	4	1.769	0.367	0.512–6.108						
C*04:06:01	3	0	–	–	–						
C*05:01:01	0	2	–	–	–						
C*06:02:01	20	4	0.661	0.469	0.216–2.022						
C*07:01:01	2	1	1.727	0.658	0.153–19.408						
C*07:02:01	54	17	1.113	0.745	0.581–2.135						
C*07:02:99	1	0	–	–	–						
C*07:04:01	15	6	1.416	0.494	0.522–3.839						
C*07:06:01	26	8	1.064	0.887	0.452–2.502						
C*07:18:01	0	1	–	–	–						
C*08:01:01	36	7	0.619	0.280	0.259–1.478						
C*08:22:01	4	1	0.854	0.889	0.093–7.803						
C*12:02:02	12	2	0.555	0.451	0.120–2.559						
C*12:03:01	10	0	–	–	–						
C*14:02:01	**10**	**9**	**3.485**	**0.010**	**1.339–9.065**						
C*15:02:01	20	6	1.032	0.949	0.393–2.709						
C*15:05:02	2	0	–	–	–						
C*18:02:01	1	0	–	–	–						

Significance different *P*-value < 0.05; *HLA-A* *human leukocyte antigen-A*; *HLA-B* *human leukocyte antigen-B*; *HLA-C human leukocyte antigen-C*; *BD* Behcet’s disease; Odds ratio, 95%CI, 95% Confidence interval, P-value were calculated using Fisher’s exact testing; Data analysis result was presented statistical significance (*p*-value < 0.05).

**Table 3:** Analysis of various *HLA* Class II molecules and BD associations using NGS.

*HLA-DPB1* 6-Digit	Control BD n = 192, n (%)	BD n = 56, n (%)	Odds Ratio	*P*-value	95%CI	*HLA-DRB1* 6-Digit	Control BD n = 192, n (%)	BD n = 56, n (%)	Odds Ratio	*P*-value	95%CI
DPB1*01:01:01	9 (4.69)	4 (7.14)	1.564	0.471	0.462–5.284	DRB1*01:01:01	3 (1.56)	0 (0.00)	–	–	–
DPB1*02:01:02	53 (27.60)	12 (21.43)	0.715	0.357	0.350–1.458	DRB1*03:01:01	14 (7.29)	2 (3.57)	0.470	0.329	0.103–2.137
DPB1*02:02:01	26 (13.54)	10 (17.86)	1.387	0.421	0.624–9.086	DRB1*03:02:01	21 (10.94)	2 (3.57)	0.301	0.113	0.068–1.327
DPB1*03:01:01	26 (13.54)	6 (10.71)	0.766	0.580	0.298–1.965	DRB1*04:03:01	3 (1.56)	0 (0.00)	–	–	–
DPB1*04:01:01	37 (19.27)	7 (12.50)	0.598	0.247	0.250–1.427	DRB1*04:05:01	2 (1.04)	0 (0.00)	–	–	–
DPB1*04:02:01	20 (10.42)	3 (5.36)	0.486	0.260	0.139–1.702	DRB1*07:01:01	24 (12.50)	10 (17.86)	1.521	0.308	0.679–3.409
DPB1*05:01:01	**71 (36.98)**	**30 (53.57)**	**1.966**	**0.028**	**1.077–3.587**	DRB1*08:02:01	0 (0.00)	2 (3.57)	–	–	–
DPB1*06:01:01	1 (0.52)	0 (0.00)	–	–	–	DRB1*08:03:02	14 (7.29)	2 (3.57)	0.470	0.329	0.103–2.137
DPB1*09:01:01	9 (4.69)	1 (1.79)	0.369	0.350	0.045–2.982	DRB1*09:01:02	17 (8.85)	2 (3.57)	0.381	0.207	0.085–1.702
DPB1*10:01:01	5 (2.60)	0 (0.00)	–	–	–	DRB1*10:01:01	16 (8.33)	1 (1.79)	0.200	0.123	0.025–1.542
DPB1*13:01:01	48 (25.00)	16 (28.57)	1.200	0.591	0.616–2.334	DRB1*11:01:01	55 (28.65)	19 (33.93)	1.279	0.448	0.677–2.414
DPB1*14:01:01	15 (7.81)	1 (1.79)	0.214	0.140	0.027–1.661	DRB1*11:03:01	4 (2.08)	0 (0.00)	–	–	–
DPB1*16:01:01	1 (0.52)	2 (3.57)	–	–	–	DRB1*11:04:01	2 (1.04)	0 (0.00)	–	–	–
DPB1*17:01:01	8 (4.17)	5 (8.93)	2.254	0.169	0.707–7.190	DRB1*12:01:01	4 (2.08)	2 (3.57)	1.740	0.529	0.310–9.761
DPB1*19:01:01	3 (1.56)	2 (3.57)	2.333	0.360	0.380–14.323	DRB1*12:02:01	34 (17.71)	15 (26.79)	1.700	0.136	0.846–3.416
DPB1*21:01	6 (3.13)	3 (5.36)	1.754	0.437	0.424–7.252	DRB1*13:01:01	1 (0.52)	1 (1.79)	3.472	0.381	0.213–56.425
DPB1*22:01:01	3 (1.56)	1 (1.79)	1.145	0.907	0.116–11.232	DRB1*13:02:01	4 (2.08)	1 (1.79)	0.854	0.889	0.093–7.803
DPB1*23:01:01	0 (0.00)	1 (1.79)	–	–	–	DRB1*13:07:01	6 (3.13)	0 (0.00)	–	–	–
DPB1*26:01:02	4 (2.08)	2 (3.57)	1.740	0.529	0.310–9.761	DRB1*14:02:01	14 (7.29)	3 (5.36)	0.719	0.616	0.199–2.599
DPB1*31:01:01	4 (2.08)	1 (1.79)	0.854	0.889	0.093–7.803	DRB1*14:04:01	1 (0.52)	1 (1.79)	3.472	0.381	0.213–56.425
DPB1*34:01:01	1 (0.52)	0 (0.00)	–	–	–	DRB1*14:05:01	24 (12.50)	12 (21.43)	1.909	0.099	0.885–4.116
DPB1*38:01	0 (0.00)	1 (1.79)	–	–	–	DRB1*14:06:01	5 (2.60)	0 (0.00)	–	–	–
DPB1*39:01:01	3 (1.56)	0 (0.00)	–	–	–	DRB1*14:07:01	1 (0.52)	0 (0.00)	–	–	–
DPB1*47:01:01	3 (1.56)	0 (0.00)	–	–	–	DRB1*14:54:01	**82 (42.71)**	**33 (58.93)**	**1.924**	**0.034**	**1.051–3.522**
DPB1*48:01	1 (0.52)	0 (0.00)	–	–	–	DRB1*15:01:01	4 (2.08)	0 (0.00)	–	–	–
						DRB1*15:02:01	20 (10.42)	2 (3.57)	0.318	0.131	0.072–1.406
						DRB1*16:02:01	7 (3.65)	1 (1.79)	0.480	0.497	0.057–3.990

*HLA-DQB1* 6-Digit	Control BD n = 192, n (%)	BD n = 56, n (%)	Odds Ratio	*P*-value	95%CI						
DQB1*02:01:01	9 (4.69)	2 (3.57)	0.753	0.722	0.157–3.590						
DQB1*02:02:01	29 (15.10)	8 (14.29)	0.936	0.880	0.401–2.183						
DQB1*03:01:01	75 (39.06)	22 (39.29)	1.009	0.976	0.548–1.857						
DQB1*03:02:01	29 (15.10)	2 (3.57)	0.208	0.036	0.048–0.901						
DQB1*03:03:02	41 (21.35)	7 (12.50)	0.526	0.145	0.221–1.248						
DQB1*04:01:01	1 (0.52)	0 (0.00)	–	–	–						
DQB1*04:02:01	16 (8.33)	4 (7.14)	0.846	0.774	0.271–2.641						
DQB1*05:01:01	20 (10.42)	1 (1.79)	0.156	0.073	0.020–1.191						
DQB1*05:01:24	29 (15.10)	5 (8.93)	0.551	0.243	0.202–1.497						
DQB1*05:02:01	57 (29.69)	19 (33.93)	1.216	0.545	0.645–2.292						
DQB1*05:03:01	**16 (8.33)**	**12 (21.43)**	**3.000**	**0.008**	**1.323–6.798**						
DQB1*06:01:01	23 (11.98)	7 (12.50)	1.049	0.916	0.425–2.591						
DQB1*06:02:01	3 (1.56)	2 (3.57)	2.333	0.360	0.380–14.323						
DQB1*06:03:01	2 (1.04)	1 (1.79)	1.727	0.658	0.153–19.408						
DQB1*06:04:01	1 (0.52)	0 (0.00)	–	–	–						
DQB1*06:09:01	4 (2.08)	2 (3.57)	0.854	0.889	0.093–7.803						

Significance different *P*-value < 0.05; *HLA-DPB1* *human leukocyte antigen-DPB1*;* HLA-DRB1* *human leukocyte antigen-DRB1*; *HLA-DQB1* *human leukocyte antigen-DQB1*; *BD* Behcet’s disease; Odds ratio, 95%CI, 95% Confidence interval, P-value were calculated using Fisher’s exact testing; Data analysis result was presented statistical significance (*p*-value < 0.05).

### Haplotype analysis

To elucidate the association among different *HLA* alleles across multiple loci, pairwise haplotype analysis was conducted. The analysis identified a linkage disequilibrium in the haplotypes HLA-A*24:02:01, HLA-B*51:01:01, and HLA-C*14:02:02 (A–B–C, *P*-value = 0.033, Pc-value = 0.066). Subsequent pairwise haplotype analyses were performed, revealing a significant correlation in the B–C pair (HLA-B*51:01:01; HLA-C*14:02:01, *P*-value = 0.01, Pc-value = 0.02) but not in the A–C pair (HLA-A*24:02:01, HLA-C*14:02:01, *P*-value = 0.45).

### Susceptible *HLA* alleles associated with the ocular/vascular phenotype of Behcet’s disease (BD)

The alleles linked to patients with BD exhibiting ocular involvement were investigated in this study. Among the various reported alleles, the HLA-B*51:01 allele emerged as the most significant risk allele associated with BD patients with ocular manifestations (*p* = 0.02). At the 6-digit resolution, a noteworthy association was observed between the HLA-DRB1*14:54:01 (OR 11.67, 95% CI 2.86–47.57, *p* = 0.001) and BD patients with ocular involvement. Conversely, individuals with BD and vascular complications exhibited a higher prevalence of HLA-DRB1*14:54:01 alleles (OR 3.352, 95% CI 1.00–11.19, *p* = 0.049). However, it is found to be statistically insignificant.

## Discussion

This study employed six-digit genotyping to discern risk alleles associated with Behçet's disease (BD) patients, focusing on susceptible allele subtypes and specific phenotypes such as ocular and vascular involvement in Thai BD patients. Our findings of univariate analysis underscore HLA-B*51:01:02, HLA-B*39:01:01, HLA-C*14:02:01, and HLA-DQB1*05:03:01 alleles as the primary risk alleles in BD patients, a correlation consistent with numerous earlier studies across diverse ethnicities. HLA-B*51:01 emerges as the predominant allele associated with BD across diverse ethnicities, underscoring its pivotal role in BD susceptibility. The novel discovery of HLA-B*51 subtypes reflect the genetic diversity within this allele group, suggesting a complex landscape of variations. Recent advancements in genetic research have led to the identification of novel variants such as HLA-B*51:94, HLA-B*51:151, and HLA-B*51:220, each characterized by distinct nucleotide substitutions. Notably, subtype HLA-B*51:08 exhibits amino acid variations compared to HLA-B*51:01, particularly at positions 152 and 156 within pocket E of the *HLA* molecule. These unique amino acid signatures differentiate HLA-B*51:08 from other subtypes and may influence its association with BD risk. Together, these findings underscore the intricate genetic architecture of HLA-B*51 alleles and their significance in BD susceptibility across different populations^27^. Notably, a genome-wide association study (GWAS) conducted in Spain identified independent risk alleles, including HLA-B*51:01 and HLA-A*26:01^14^. Similarly, studies in Turkey and Saudi Arabia confirmed HLA-B*51:01 as a predominant genetic marker in BD patients^29,30^ Greek research identified the involvement of the *MICA-TM A6* allele and HLA-B*51:01 in Behçet's Disease within a European population. Furthermore, research conducted in Spain, comprising 278 BD patients and 1517 healthy individuals, consistently emphasized the significant association of HLA-B*51:01 and HLA-A*03:01. These results were subsequently validated in multiple studies within the same population. The presence of HLA-B*51:01 has also been observed in the Argentinian BD population^26^, reinforcing its status as the predominant allele in BD patients, irrespective of ethnic background or clinical phenotype. In this study, further subtype analysis of HLA-B*51:01 revealed HLA-B*51:01:02 as the most significant subtype associated with BD. However, the Bonferroni’s correction (multiple variable analysis) we have performed in this study, hasn’t found any association between HLA-B*51:01:02, and BD. This could be attributed to the limited sample size or high data variability, leading to associations observed in univariate analysis failing to reach significance in multivariable analysis.

Nevertheless, achieving a comprehensive understanding of BD diagnosis requires examination of *HLA* and non-*HLA* genetic variants, as well as consideration of environmental factors. This is particularly crucial in populations lacking the most common allele, HLA-B*51:01. Previous studies have reported numerous risk alleles, both within the *HLA* system and beyond. In Thailand, an in-silico analysis revealed the strongest binding affinity for HLA-B*51:01, followed by HLA-B*35:01, HLA-A*26:01, and HLA-A*11:01^27^. A Korean study, encompassing 223 BD patients and 1398 healthy controls, observed a higher prevalence of the HLA-A*02:07, HLA-A*26:01, and HLA-A*30:04 alleles in BD patients compared to controls^19^. Notably, this association had odds of 4.19 or greater among patients lacking the HLA-B*51 allele. In Japanese study, HLA-A*26:01 was identified as a risk allele, particularly noteworthy in patients lacking the HLA-B*51:01 allele, and their findings suggested an association with a poor prognosis^28^. This observation was consistently reported in another GWAS conducted within the same population^29^. In Middle Eastern countries, Saudi Arabia specifically reported HLA-A*26:01 as a risk allele, alongside others such as HLA-B*51:01 and HLA-A*31 associated with BD^22^. While HLA-A*26:01 has frequently been identified as a predisposing allele for BD across various populations, in this study, although initially significant, this association lost statistical significance following Bonferroni correction for type 1 error.

In the European population, HLA-B*27 and its subtypes have been linked to BD. Alireza Khabbazi et al.^31^ conducted a meta-analysis study, establishing a significant relationship between HLA-B*27 and BD across various populations. Ethnic variations were noted, with the prevalence of the HLA-B*27 allele being higher in European populations^30^. Another meta-analysis study by Capittini et al.^32^ encompassing diverse global populations, reported HLA-DQB1*03 and HLA-A*26, in addition to HLA-B*51:01 and HLA-B*51:02, as genetic factors associated with BD.

Although previous studies did not find any association between HLA-B*39:01:01 and BD in Thai and other populations, a study conducted in Japan reported this allele's association with familial Mediterranean fever (FMF) in Japanese patients^33^. Haplotype analysis and linkage disequilibrium are critical in *HLA* allele and disease association studies. The high genetic diversity within the *HLA* region necessitates an understanding of how specific alleles are inherited together on the same chromosome. In *HLA* studies, these analyses offer precision in identifying risk alleles, shedding light on the complex genetic basis of diseases, and facilitating targeted therapeutic approaches for personalized medicine. The results of our pairwise haplotype analysis shed light on the intricate associations among different *HLA* alleles in the context of BD. Notably, the analysis revealed a significant linkage disequilibrium in the HLA-A*24:02:01, HLA-B*51:01:01, HLA-C*14:02:02 haplotypes (A–B–C, *P*-value = 0.033, PC-value = 0.066), emphasizing the coinheritance of specific alleles on the same chromosome. This haplotype was reported in one of the Indian studies, especially in south Indians, with a haplotype frequency of 0.57%^34^. The same haplotype was reported in *HLA* allele database in the Chinese in Chinese Jingpo minority at 1.04%^35^. However, this is the first time in the Thai population that this haplotype has been found to be associated in BD patients. Further exploration of pairwise haplotypes led to the identification of a noteworthy correlation within the B–C pair (HLA-B*51:01:01; HLA-C*14:02:01, *P*-value = 0.01, Pc-value = 0.02). This finding suggests a potential synergistic effect or shared genetic influence between the HLA-B*51:01:01 and HLA-C*14:02:01 alleles in BD. Therefore, when we considered with HLA-B*51:01:01 (OR = 3.033, 95% CI = 1.135–8.103, *P*-value = 0.027) and HLA-C*14:02:01(OR = 3.485, 95% CI = 1.339–9.065, *P*-value = 0.001) were associated with BD. HLA-B*51:01:01 can be useful for screening marker in Thai population. In contrast, the A–C pair (HLA-A*26:01:01, HLA-C*03:02:02) did not exhibit a statistically significant correlation (*P*-value = 0.45), suggesting that the observed linkage disequilibrium may not extend to these particular alleles or that other factors influence their inheritance patterns. These results underscore the complexity of *HLA* associations in BD and highlight the importance of considering specific allele combinations in understanding the genetic basis of the disease. A worldwide meta-analysis investigating the link between BD and variations in genes of both *HLA* Class I (A, B, and C) and Class II (DRB1, DQB1, and DPB1) has confirmed that the *HLA-B ∗ 51;Cw ∗ 15* and *HLA-B ∗ 51*;*Cw ∗ 14* haplotypes were the second and third most frequent haplotypes, respectively, while the *HLA-B ∗ 51*; *Cw ∗ 16* haplotype was in sixth place^32^. Further studies with larger cohorts and diverse populations are warranted to validate and extend these findings, providing additional insights into the genetic architecture of BD.

This study's primary limitation was its small sample size, limiting exploration of *HLA* relationships with CVS, GI, and neurological phenotypes. The exclusive focus was on the association between *HLA-B* alleles and BD, excluding a broader analysis of all *HLA* alleles or other phenotypes.

## Conclusion

Our research highlights specific *HLA* associations in Behçet's disease (BD), particularly the significant relation of HLA-DRB1*14:54:01 with ocular manifestations. Comprehensive haplotype analysis revealed a correlation between HLA-B*51:01:01 and HLA-C*14:02:01 suggests a potential synergistic effect in BD. These insights underscore the complexity of *HLA* associations in BD and stress the importance of considering specific allele combinations for a nuanced understanding. When we considered association between this allele and BD. Therefore, HLA-B*51:01:01 can be useful for screening marker in Thai population. Further studies with diverse populations are crucial for validating these findings and advancing targeted therapeutic approaches in personalized medicine.

## Material and methods

### Study population

Patients were recruited at Mahidol University's Faculty of Medicine and Ramathibodi Hospital's dermatology and ophthalmology clinics between 2013 and 2018. Patients who scored greater than or equal to four on the ICBD^36^ criteria and were diagnosed with BD by a rheumatologist and a dermatologist were included in this study. Overall, 56 BD patients were enrolled in the study. Their clinical characteristics and sociodemographic details were obtained from medical records. This study includes a control group from the Electricity Generating Authority of Thailand (EGAT) project.

### Genomic DNA extraction

Blood samples were taken in EDTA tubes. DNA was isolated using magnetic-bead technology on the Roche Diagnostics, USA, MagNA Pure Compact automated extraction equipment. The genomic DNA's quality and quantity were analysed using Nano Drop (ND-1000). All DNA was aliquoted and stored at − 20 °C before analysis. The quantity (concentration) of DNA was measured by Qubit® Fluorometer 2.0 (Life Technologies) with Qubit® dsDNA HS Assay Kits (Life Technologies), and the quality (size) of DNA was run with 100–200 mg of known DNA on 1% agarose gel electrophoresis compared with O’GeneRuler 1 kb DNA Ladder (Thermo Scientific). DNA was selected if the concentration is over or equal to 25 ng/µl and the size of DNA was more than 10 kb.

### *HLA* genotyping

*HLA* class I (*HLA-A, -B, C*) and *HLA* class II (*HLA-DRB1, -DQB1, DPB1*) amplicons were generated using the NXTypeTM reagents. And the amplicons were amplified according to One Lambda, USA protocol for PCR amplicons greater than 3 kb. Afterwards, the amplicons underwent quantity check using Qubit® dsDNA HS Assay Kits (Life Technologies), and DNA 12K assay, DNA Extended Range Chip (12K) (PerkinElmer, USA).

*HLA* class I and class II amplicons (6-types) were pooled together in equimolar proportions and subsequently purified with AMPure® PB (Pacific Biosciences, USA). SMRTbell libraries were then generated using the SMRTbell Barcoded Adapter Complete Prep kit–96 (Pacific Biosciences, USA). Individual samples, consisting of pooled *HLA* amplicons, were end-repaired, and ligated to unique SMRTbell barcoded adapters in a single reaction. Following ligation, the libraries were pooled and further purified using AMPure® PB. The resulting purified libraries were prepared for sequencing with the Sequel Binding and Internal Control Kit 3.0 (Pacific Biosciences, USA) and were subsequently sequenced on PacBio Sequel System (Pacific Bioscience, USA). Sequencing was performed using the Sequel sequencing kit 3.0 and SMRTcell 1M v3 (Pacific Bioscience, USA) with 10 h movie time.

Demultiplexed, high-quality consensus sequences (Long Amplicon Analysis; LAA reads) of each sample were generated in FastQ from raw sequence data using the accompanying analysis software suite, SMRT Link version 9.0, with given parameters of at least ten passes and 99.9% accuracy.

For downstream analysis, a custom full-length *HLA* reference alleles was created from *HLA* class I (*HLA-A, -B, -C*) and *HLA* class II (*HLA-DRB1, -DQB1, -DPB1*) reference allele sequences (IMGT/HLA release 3.38.0). Due to lack of standardized analysis guidelines for long-read *HLA* data, custom analysis pipeline was used (“Supplementary Information”).

Briefly, for each sample, the consensus LAA reads were aligned against the custom full-length *HLA* reference alleles using minimap2 version 2.8^37^. From the alignment outputs, numbers of LAA reads fully mapped to each *HLA* alleles are generated and the list of ‘hit’ *HLA* alleles for each *HLA* gene was sorted according to highest numbers of mapped LAA reads. For each sample, a maximum of four candidate *HLA* alleles per *HLA* gene were selected based on the highest numbers of mapped LAA reads and at least 50 mapped LAA reads. In most cases, there were two candidate *HLA* alleles (sometimes, only one) per *HLA* gene per sample with much higher number of mapped LAA reads. These *HLA* alleles were then assigned as the *HLA* alleles within the sample (*HLA* genotyping).

To assure the accuracy of assigned *HLA* alleles whether each assigned *HLA* allele was a known *HLA* allele or probably a novel *HLA* allele, a representative consensus sequence obtained from the LAA sequences matching each candidate *HLA* allele was aligned against the current version of reference *HLA* alleles in IMGT/HLA database, using the IPD-IMGT/HLA Sequence Alignment Tool on the EBI website; https://www.ebi.ac.uk/ipd/imgt/hla/alignment/. The finalized candidate *HLA* alleles were assigned to each sample for each of the six *HLA* class I and class II genes.

### Statistical analysis

*HLA* alleles were tested for their association with BD by calculating the Odds ratio; 95% Confidence Interval using Fisher’s exact test. STATA version 12 for Windows was used to analyses all tests. The corrected P-values (Pc-value) for multiple comparison of *HLA* alleles (34 for *HLA-A*, 49 for *HLA-B*, 24 for *HLA-C*, 25 for *HLA-DPB1*, 27 for *HLA-DRB1*, and 16 for *HLA-DQB1*) were calculated using Bonferroni’s correction. *P*-value were less than 0.05 was considered statistically significant. To reduce the likelihood of type 1 error, Bonferroni correction was applied. Following this correction, a significance threshold of *p* = 0.01 (< 0.05/4—two-tailed) was adopted. Haplotype association analysis was carried out using ‘haplo.stats’ R Studio software packages.

### Ethics approval

Our study strictly adheres to the Declaration of Helsinki's principles. Participants receive a clear and understandable patient information sheet along with a comprehensive informed consent form before participating. We exclude vulnerable groups (children, pregnant women, prisoners, and those with diminished decision-making capacity). Our research protocol underwent rigorous ethical review by an accredited board, which approved it based on the highest ethical standards by the Committee for Research, Faculty of Medicine Ramathibodi Hospital Mahidol University (COA. MURA2020/16).

### Supplementary Information

Supplementary Information.

## Data Availability

The raw data supporting the conclusions of this article will be made availability by the C.S. without undue reservation.
