# Supplementary material for: Novel genetic variants of HLA gene associated with Thai Behcet’s disease (BD) patients using next generation sequencing technology
Source: Sci Rep. 2024 Apr 4;14:7967. doi: 10.1038/s41598-024-58254-w (PMC10995258; doi:10.1038/s41598-024-58254-w)
Supplement: Supplementary file 1 — Supplementary Information. [file 41598_2024_58254_MOESM1_ESM.pdf]

Novel Genetic Variants of HLA Gene Associated with Thai Behcet's Disease (BD) Patients Using Next Generation Sequencing Technology.

Supplement 1 : HLA genotyping of Behcet's and Control

TypeN0	HLA-A	HLA-B	HLA-C	HLA-DPB1	HLA-DQB11	HLA-RB1
BD04	*A*11:01:01 / A*24:02:01*	*B*39:01:01 / B*51:01:01*	*C*15:02:01 / C*07:02:01*	*DPB1*19:01:01 / DPB1*02:01:02*	*DQB1*05:02:01 / DQB1*02:02:01*	*DRB1*07:01:01 / DRB1*14:54:01*
BD05	*A*11:01:75 / A*26:01:01*	*B*13:01:01 / B*44:02:01*	*C*04:03:01 / C*05:01:01*	*DPB1*02:01:02 / DPB1*04:01:01*	*DQB1*05:03:01 / DQB1*06:03:01*	*DRB1*14:04:01 / DRB1*13:01:01*
BD06	*A*02:03:01 / A*11:01:01*	*B*40:01:02 / B*51:01:01*	*C*14:02:01 / C*03:04:01*	*DPB1*05:01:01 / DPB1*13:01:01*	*DQB1*05:02:01 / DQB1*03:03:02*	*DRB1*11:01:01 / DRB1*09:01:02*
BD07	*A*02:07:01 / A*11:01:01*	*B*46:01:01 / B*15:07:01*	*C*01:02:01 / C*01:02:01*	*DPB1*02:02:01 / DPB1*05:01:01*	*DQB1*05:02:01 /DQB1*03:03:02*	*DRB1*14:05:01 / DRB1*14:54:01*
BD09	*A*11:01:01 / A*24:02:01*	*B*18:01:01 / B*07:02:01*	*C*07:04:01 / C*07:02:01*	*DPB1*13:01:01 / DPB1*13:01:01*	*DQB1*05:01:24 / DQB1*03:01:01*	*DRB1*12:02:01 / DRB1*11:01:01*
BD10	*A*24:03:01 / A*24:10:01*	*B*13:01:01 / B*18:01:01*	*C*07:04:01 / C*03:04:01*	*DPB1*21:01 / DPB1*05:01:01*	*DQB1*04:02:01 / DQB1*05:03:01*	*DRB1*14:05:01 / DRB1*14:54:01*
BD12	*A*02:11:01 / A*33:03:01*	*B*44:03:02 / B*44:03:02*	*C*07:06:01 / C*07:18:01*	*DPB1*13:01:01 / DPB1*01:01:01*	*DQB1*06:02:01 / DQB1*02:02:01*	*DRB1*11:01:01 / DRB1*07:01:01*
BD16	*A*11:01:01 / A*33:03:01*	*B*40:01:02 / B*44:03:02*	*C*03:04:01 / C*07:06:01*	*DPB1*13:01:01 / DPB1*05:01:01*	*DQB1*05:02:01 / DQB1*05:02:01*	*DRB1*11:01:01 / DRB1*03:02:01*
BD17	*A*29:01:01 / A*30:01:01*	*B*13:02:01 / B*52:01:01*	*C*04:03:01 / C*12:02:02*	*DPB1*17:01:01 / DPB1*04:01:01*	*DQB1*06:01:01 / DQB1*02:02:01*	*DRB1*07:01:01 / DRB1*11:01:01*
BD18	*A*24:02:01 / A*33:03:01*	*B*15:25:01 / B*44:03:02*	*C*07:02:01 / C*07:06:01*	*DPB1*19:01:01 / DPB1*04:02:01*	*DQB1*03:01:01 / DQB1*02:02:01*	*DRB1*12:02:01 / DRB1*14:54:01*
BD19	*A*11:01:01 / A*24:10:01*	*B*18:01:01 / B*51:01:01*	*C*07:04:01 / C*15:02:01*	*DPB1*02:02:01 / DPB1*05:01:01*	*DQB1*05:03:01 / DQB1*03:01:01*	*DRB1*14:54:01 / DRB1*12:02:01*
BD20	*A*11:01:01 / A*33:03:01*	*B*58:01:01 / B*52:01:01*	*C*03:02:02 / C*07:02:01*	*DPB1*13:01:01 / DPB1*04:01:01*	*DQB1*03:01:01 / DQB1*02:01:01*	*DRB1*14:05:01 / DRB1*03:01:01*
BD21	*A*02:07:01 / A*02:07:01*	*B*07:02:01 / B*46:01:01*	*C*01:02:01 / C*07:02:01*	*DPB1*05:01:01 / DPB1*03:01:01*	*DQB1*03:03:02 / DQB1*03:03:02*	*DRB1*14:02:01 / DRB1*03:02:01*
BD22	*A*02:07:01 / A*33:03:01*	*B*44:03:02 / B*46:01:01*	*C*01:02:01 / C*07:01:01*	*DPB1*21:01 / DPB1*02:01:02*	*DQB1*05:02:01 / DQB1*05:02:01*	*DRB1*12:02:01 / DRB1*14:54:01*
BD24	*A*24:02:01 / A*26:01:01*	*B*18:01:01 / B*40:01:02*	*C*07:04:01 / C*03:04:01*	*DPB1*05:01:01 / DPB1*03:01:01*	*DQB1*06:02:01 / DQB1*03:01:01*	*DRB1*11:01:01 / DRB1*14:54:01*
BD25	*A*26:01:01 / A*24:02:01*	*B*15:02:01 / B*40:02:01*	*C*01:02:01 / C*08:01:01*	*DPB1*05:01:01 / DPB1*01:01:01*	*DQB1*05:02:01 / DQB1*05:03:01*	*DRB1*12:02:01 / DRB1*14:54:01*
BD26	*A*02:07:01 / A*24:07:01*	*B*15:02:01 / B*46:01:01*	*C*01:02:01 / C*08:01:01*	*DPB1*04:01:01 / DPB1*13:01:01*	*DQB1*06:01:01 / DQB1*05:02:01*	*DRB1*11:01:01 / DRB1*14:54:01*
BD27	*A*02:01:01 / A*33:03:01*	*B*15:02:01 / B*44:03:02*	*C*07:06:01 / C*08:01:01*	*DPB1*31:01:01 / DPB1*02:01:02*	*DQB1*03:02:01 / DQB1*03:02:01*	*DRB1*07:01:01 / DRB1*14:02:01*
BD28	*A*11:01:01 / A*11:01:01*	*B*13:01:01 / B*51:01:02*	*C*14:02:01 / C*03:04:01*	*DPB1*02:02:01 / DPB1*05:01:01*	*DQB1*05:03:01 / DQB1*05:02:01*	*DRB1*14:05:01 / DRB1*14:54:01*
BD29	*A*24:02:01 / A*24:02:01*	*B*39:01:01 / B*44:03:02*	*C*07:02:01 / C*07:06:01*	*DPB1*13:01:01 / DPB1*05:01:01*	*DQB1*02:02:01 / DQB1*05:02:01*	*DRB1*14:54:01 / DRB1*07:01:01*
BD30	*A*02:03:01 / A*33:03:01*	*B*44:03:02 / B*44:03:01*	*C*07:06:01 / C*07:06:01*	*DPB1*02:02:01 / DPB1*04:02:01*	*DQB1*02:02:01 / DQB1*02:02:01*	*DRB1*07:01:01 / DRB1*07:01:01*
BD31	*A*11:01:01 / A*02:03:01*	*B*39:01:01 / B*56:01:01*	*C*01:02:01 / C*07:02:01*	*DPB1*13:01:01 / DPB1*05:01:01*	*DQB1*05:02:01 / DQB1*03:01:01*	*DRB1*11:01:01 / DRB1*14:54:01*
BD32	*A*11:01:01 / A*33:03:01*	*B*40:02:01 / B*44:03:02*	*C*15:02:01 / C*07:06:01*	*DPB1*13:01:01 / DPB1*05:01:01*	*DQB1*05:03:01 / DQB1*05:03:01*	*DRB1*14:05:01 / DRB1*07:01:01*
BD33	*A*11:01:01 / A*11:01:01*	*B*40:01:02 / B*55:01:01*	*C*01:02:01 / C*07:02:01*	*DPB1*05:01:01 / DPB1*05:01:01*	*DQB1*05:02:01 / DQB1*05:02:01*	*DRB1*16:02:01 / DRB1*11:01:01*
BD34	*A*11:01:01 / A*30:01:01*	*B*13:01:01 / B*13:02:01*	*C*03:04:01 / C*06:02:01*	*DPB1*17:01:01 / DPB1*05:01:01*	*DQB1*06:01:01 / DQB1*06:01:01*	*DRB1*11:01:01 / DRB1*07:01:01*
BD35	*A*26:01:01 / A*33:03:01*	*B*38:02:01 / B*58:01:01*	*C*03:02:02 / C*07:02:01*	*DPB1*02:01:02 / DPB1*14:01:01*	*DQB1*05:01:01 /DQB1*05:01:24*	*DRB1*10:01:01 / DRB1*15:02:01*
BD36	*A*11:01:01 / A*02:03:01*	*B*15:07:01 / B*39:09:01*	*C*12:02:02 / C*07:02:01*	*DPB1*13:01:01 / DPB1*04:02:01*	*DQB1*05:01:24 /DQB1*03:01:01*	*DRB1*12:02:01 / DRB1*14:54:01*
BD37	*A*02:07:01 / A*11:01:01*	*B*40:01:02 / B*46:01:01*	*C*01:02:01 / C*07:02:01*	*DPB1*13:01:01 / DPB1*03:01:01*	*DQB1*03:01:01 / DQB1*05:02:01*	*DRB1*12:02:01 / DRB1*14:54:01*
BD38	*A*11:01:01 / A*11:01:01*	*B*27:04:01 / B*51:01:01*	*C*01:02:01 / C*14:02:01*	*DPB1*03:01:01 / DPB1*05:01:01*	*DQB1*05:03:01 / DQB1*03:01:01*	*DRB1*12:01:01 / DRB1*14:54:01*
BD39	*A*02:07:01 / A*33:03:01*	*B*46:01:01 / B*51:01:02*	*C*14:02:01 / C*01:02:01*	*DPB1*02:02:01 / DPB1*05:01:01*	*DQB1*05:02:01 / DQB1*05:03:01*	*DRB1*14:05:01 / DRB1*14:54:01*
BD41	*A*24:10:01 / A*31:01:02*	*B*18:01:01 / B*46:01:01*	*C*07:04:01 / C*01:02:01*	*DPB1*01:01:01 / DPB1*03:01:01*	*DQB1*03:01:01 / DQB1*06:01:01*	*DRB1*14:54:01 / DRB1*08:03:02*
BD42	*A*33:03:01 / A*33:03:01*	*B*27:04:01 / B*44:03:02*	*C*03:04:01 / C*07:06:01*	*DPB1*17:01:01 / DPB1*05:01:01*	*DQB1*03:01:01 / DQB1*03:01:01*	*DRB1*11:01:01 / DRB1*14:54:01*
BD43	*A*02:07:01 / A*24:02:01*	*B*39:01:01 / B*46:01:01*	*C*01:02:01 / C*07:02:01*	*DPB1*05:01:01 / DPB1*04:01:01*	*DQB1*03:01:01 / DQB1*04:02:01*	*DRB1*08:02:01 / DRB1*11:01:01*
BD44	*A*26:01:01 / A*33:03:01*	*B*58:01:01 / B*58:01:01*	*C*03:02:02 / C*03:02:02*	*DPB1*02:01:02 / DPB1*04:01:01*	*DQB1*02:01:01 / DQB1*02:01:01*	*DRB1*03:01:01 / DRB1*14:05:01*
BD45	*A*01:01:01 / A*11:01:01*	*B*44:02:01 / B*54:01:01*	*C*05:01:01 / C*07:02:01*	*DPB1*02:01:02 / DPB1*13:01:01*	*DQB1*03:01:01 / DQB1*04:02:01*	*DRB1*12:02:01 / DRB1*14:54:01*
BD46	*A*02:07:01 / A*24:02:01*	*B*39:01:01 / B*46:01:01*	*C*01:02:01 / C*07:02:01*	*DPB1*05:01:01 / DPB1*23:01:01*	*DQB1*04:02:01 / DQB1*03:01:01*	*DRB1*08:02:01 / DRB1*11:01:01*
BD47	*A*02:01:01 / A*02:03:01*	*B*39:09:01 / B*40:01:02*	*C*03:04:01 / C*07:02:01*	*DPB1*05:01:01 / DPB1*05:01:01*	*DQB1*05:02:01 / DQB1*03:02:01*	*DRB1*11:01:01 / DRB1*14:02:01*
BD48	*A*11:01:01 /A*30:01:01*	*B*13:02:01 / B*56:01:01*	*C*15:02:01 / C*06:02:01*	*DPB1*01:01:01 / DPB1*26:01:02*	*DQB1*03:01:01 / DQB1*03:01:01*	*DRB1*12:02:01 / DRB1*14:54:01*
BD50	*A*11:01:01 / A*24:07:01*	*B*15:02:01 / B*46:01:01*	*C*01:02:01 / C*08:01:01*	*DPB1*02:02:01 / DPB1*05:01:01*	*DQB1*05:02:01 / DQB1*03:01:01*	*DRB1*12:02:01 / DRB1*14:54:01*
BD51	*A*02:01:01 / A*24:02:01*	*B*15:02:01 / B*51:01:01*	*C*08:01:01 / C*15:02:01*	*DPB1*21:01 / DPB1*17:01:01*	*DQB1*03:01:01 / DQB1*03:01:01*	*DRB1*12:01:01 / DRB1*14:54:01*
BD52	*A*02:07:01 / A*11:01:01*	*B*46:01:01 / B*13:01:01*	*C*01:02:01 / C*01:02:01*	*DPB1*05:01:01 / DPB1*02:01:02*	*DQB1*06:01:01 / DQB1*05:02:01*	*DRB1*14:54:01 / DRB1*11:01:01*
BD53	*A*02:03:01 / A*26:01:01*	*B*13:01:01 / B*13:01:01*	*C*03:04:01 / C*03:03:01*	*DPB1*13:01:01 / DPB1*02:02:01*	*DQB1*05:01:24 / DQB1*03:03:02*	*DRB1*11:01:01 / DRB1*09:01:02*
BD54	*A*02:07:01 / A*24:10:01*	*B*18:01:01 / B*46:01:01*	*C*07:04:01 / C*01:02:01*	*DPB1*05:01:01 / DPB1*04:01:01*	*DQB1*03:01:01 / DQB1*03:03:02*	*DRB1*14:54:01 / DRB1*12:02:01*
BD55	*A*24:02:01 / A*33:03:01*	*B*40:01:02 / B*58:01:01*	*C*03:02:02 / C*04:01:01*	*DPB1*05:01:01 / DPB1*02:02:01*	*DQB1*06:09:01 / DQB1*06:09:01*	*DRB1*14:54:01 / DRB1*13:02:01*
BD56	*A*11:01:01 / A*30:01:01*	*B*13:02:01 / B*15:01:01*	*C*03:03:01 / C*06:02:01*	*DPB1*02:02:01 / DPB1*17:01:01*	*DQB1*05:03:01 / DQB1*05:03:01*	*DRB1*14:05:01 / DRB1*07:01:01*
BD57	*A*24:02:01 / A*74:02:01*	*B*51:01:02 / B*51:01:01*	*C*14:02:01 / C*14:02:01*	*DPB1*38:01 / DPB1*05:01:01*	*DQB1*05:03:01 / DQB1*05:03:01*	*DRB1*14:54:01 / DRB1*14:05:01*
BD58	*A*02:03:01 / A*24:02:01*	*B*51:01:02 / B*51:01:01*	*C*14:02:01 / C*14:02:01*	*DPB1*13:01:01 / DPB1*05:01:01*	*DQB1*05:03:01 / DQB1*03:03:02*	*DRB1*14:54:01 / DRB1*14:05:01*
BD59	*A*02:09:01 / A*11:01:01*	*B*15:02:01 / B*15:02:01*	*C*08:01:01 / C*07:02:01*	*DPB1*05:01:01 / DPB1*05:01:01*	*DQB1*03:01:01 / DQB1*03:01:01*	*DRB1*14:54:01 / DRB1*12:02:01*
BD60	*A*02:03:01 / A*02:07:01*	*B*15:25:01 / B*46:01:19*	*C*04:03:01 / C*01:02:01*	*DPB1*22:01:01 / DPB1*05:01:01*	*DQB1*05:02:01 / DQB1*05:02:01*	*DRB1*14:54:01 / DRB1*12:02:01*
BD61	*A*02:03:01 / A*24:02:01*	*B*27:04:01 / B*40:01:02*	*C*03:04:01 / C*04:03:01*	*DPB1*13:01:01 / DPB1*13:01:01*	*DQB1*06:01:01 / DQB1*05:01:24*	*DRB1*11:01:01 / DRB1*15:02:01*
BD62	*A*24:02:01 / A*33:03:01*	*B*40:01:02 / B*51:01:01*	*C*14:02:01 / C*03:04:01*	*DPB1*02:02:01 / DPB1*03:01:01*	*DQB1*03:03:02 / DQB1*02:02:01*	*DRB1*14:54:01 / DRB1*11:01:01*
BD65	*A*02:07:01 / A*32:01:01*	*B*46:01:01 /B*40:06:01*	*C*15:02:01 / C*01:02:01*	*DPB1*02:01:02 / DPB1*05:01:01*	*DQB1*05:02:01 / DQB1*03:01:01*	*DRB1*14:05:01 / DRB1*14:54:01*
BD66	*A*24:02:01 / A*24:20:01*	*B*57:01:01 / B*51:02:02*	*C*14:02:01 / C*06:02:01*	*DPB1*02:01:02 / DPB1*02:01:02*	*DQB1*03:01:01 / DQB1*03:01:01*	*DRB1*14:54:01 / DRB1*12:02:01*
BD67	*A*24:02:01 / A*24:07:01*	*B*35:05:01 / B*15:02:01*	*C*08:01:01 / C*08:22:01*	*DPB1*26:01:02 / DPB1*02:01:02*	*DQB1*03:01:01 / DQB1*03:01:01*	*DRB1*14:54:01 / DRB1*12:02:01*
BD68	*A*02:07:01 / A*11:01:01*	*B*46:01:01 /B*44:03:02*	*C*01:02:01 / C*01:02:01*	*DPB1*02:01:02 / DPB1*05:01:01*	*DQB1*06:01:01 / DQB1*02:02:01*	*DRB1*08:03:02 / DRB1*07:01:01*
BD69	*A*11:01:01 / A*26:01:01*	*B*57:01:01 / B*51:01:02*	*C*07:02:01 / C*14:02:01*	*DPB1*09:01:01 / DPB1*13:01:01*	*DQB1*05:03:01 / DQB1*05:02:01*	*DRB1*14:05:01 / DRB1*11:01:01*
CTBD001	*A*02:07:01 / A*02:07:01*	*B*46:01:01 / B*54:01:01*	*C*01:02:01 / C*01:02:01*	*DPB1*22:01:01 / DPB1*05:01:01*	*DQB1*04:02:01 / DQB1*05:02:01*	*DRB1*14:54:01 / DRB1*12:02:01*
CTBD002	*A*11:01:01 / A*11:01:01*	*B*15:18:01 / B*40:01:02*	*C*07:04:01 / C*07:02:01*	*DPB1*13:01:01 / DPB1*03:01:01*	*DQB1*05:01:24 / DQB1*05:02:01*	*DRB1*11:01:01 / DRB1*14:05:01*
CTBD003	*A*34:01:01 / A*24:02:01*	*B*35:05:01 / B*40:02:01*	*C*15:02:01 / C*04:01:01*	*DPB1*02:02:01 / DPB1*48:01*	*DQB1*06:01:01 / DQB1*03:01:01*	*DRB1*14:54:01 / DRB1*11:01:01*
CTBD004	*A*11:01:01 / A*33:03:01*	*B*58:01:01 / B*52:01:01*	*C*03:02:02 / C*07:02:01*	*DPB1*01:01:01 / DPB1*04:02:01*	*DQB1*03:01:01 / DQB1*02:01:01*	*DRB1*03:01:01 / DRB1*14:05:01*
CTBD005	*A*74:02:01 / A*24:10:01*	*B*18:01:01 / B*18:01:01*	*C*07:04:01 / C*07:04:01*	*DPB1*01:01:01 / DPB1*13:01:01*	*DQB1*03:01:01 / DQB1*03:01:01*	*DRB1*14:54:01 / DRB1*12:02:01*
CTBD006	*A*24:07:01 / A*33:03:01*	*B*46:01:01 / B*07:02:01*	*C*01:02:01 / C*07:02:01*	*DPB1*21:01 / DPB1*13:01:01*	*DQB1*05:01:24 / DQB1*05:02:01*	*DRB1*14:54:01 / DRB1*15:02:01*
CTBD007	*A*02:07:01 / A*24:10:01*	*B*46:01:01 / B*18:01:01*	*C*07:04:01 / C*01:02:01*	*DPB1*14:01:01 / DPB1*03:01:01*	*DQB1*03:03:02 / DQB1*03:01:01*	*DRB1*14:54:01 / DRB1*12:01:01*
CTBD008	*A*11:01:01 / A*26:01:01*	*B*40:06:01 / B*51:01:01*	*C*14:02:01 / C*03:04:01*	*DPB1*13:01:01 / DPB1*02:01:02*	*DQB1*06:01:01 / DQB1*03:03:02*	*DRB1*08:03:02 / DRB1*09:01:02*
CTBD009	*A*02:03:01 / A*33:03:01*	*B*55:01:01 / B*58:01:01*	*C*03:02:02 / C*12:03:01*	*DPB1*19:01:01 / DPB1*04:01:01*	*DQB1*02:01:01 / DQB1*02:02:01*	*DRB1*14:05:01 / DRB1*03:01:01*
CTBD010	*A*33:03:01 / A*33:03:01*	*B*44:03:02 / B*18:01:01*	*C*07:06:01 / C*07:04:01*	*DPB1*02:01:02 / DPB1*03:01:01*	*DQB1*03:02:01 / DQB1*02:02:01*	*DRB1*03:02:01 / DRB1*07:01:01*
CTBD011	*A*02:03:01 / A*31:01:02*	*B*46:01:01 / B*51:02:01*	*C*15:02:01 / C*01:02:01*	*DPB1*13:01:01 / DPB1*02:02:01*	*DQB1*03:01:01 / DQB1*03:01:01*	*DRB1*14:54:01 / DRB1*12:02:01*
CTBD012	*A*02:06:01 / A*02:01:01*	*B*40:06:01 / B*15:02:01*	*C*08:01:01 / C*08:22:01*	*DPB1*02:01:02 / DPB1*04:01:01*	*DQB1*03:01:01 / DQB1*03:02:01*	*DRB1*14:54:01 / DRB1*12:02:01*
CTBD013	*A*02:07:01 / A*11:01:01*	*B*46:01:01 / B*46:01:01*	*C*01:02:01 / C*01:02:01*	*DPB1*02:02:01 / DPB1*03:01:01*	*DQB1*03:03:02 / DQB1*03:01:01*	*DRB1*11:01:01 / DRB1*09:01:02*
CTBD014	*A*02:03:01 / A*30:01:01*	*B*13:02:01 / B*13:01:01*	*C*04:03:01 / C*06:02:01*	*DPB1*17:01:01 / DPB1*02:01:02*	*DQB1*05:01:24 / DQB1*02:02:01*	*DRB1*11:01:01 / DRB1*15:02:01*

Novel Genetic Variants of HLA Gene Associated with Thai Behcet’s Disease (BD) Patients Using Next Generation Sequencing Technology.

Supplement 1 : HLA genotyping of Behcet's and Control

TypeN0	HLA-A	HLA-B	HLA-C	HLA-DPB1	HLA-DQB11	HLA-RB1
CTBD015	*A*24:02:01 / A*24:02:01*	*B*40:01:02 / B*56:01:01*	*C*01:02:01 / C*03:04:01*	*DPB1*13:01:01 / DPB1*04:01:01*	*DQB1*06:01:01 / DQB1*03:03:02*	*DRB1*08:03:02 / DRB1*09:01:02*
CTBD016	*A*11:01:01 / A*11:02:01*	*B*46:01:01 / B*40:01:02*	*C*01:02:01 / C*07:02:01*	*DPB1*05:01:01 / DPB1*05:01:01*	*DQB1*03:03:02 / DQB1*03:01:01*	*DRB1*11:01:01 / DRB1*09:01:02*
CTBD017	*A*02:11:01 / A*33:03:01*	*B*44:03:02 / B*58:01:01*	*C*07:06:01 / C*03:02:02*	*DPB1*04:01:01 / DPB1*04:02:01*	*DQB1*02:01:01 / DQB1*02:02:01*	*DRB1*03:01:01 / DRB1*14:05:01*
CTBD018	*A*33:03:01 / A*11:01:01*	*B*27:04:01 / B*58:01:01*	*C*03:02:02 / C*12:02:02*	*DPB1*02:02:01 / DPB1*19:01:01*	*DQB1*05:03:01 / DQB1*02:01:01*	*DRB1*14:05:01 / DRB1*03:01:01*
CTBD019	*A*02:03:01 / A*11:01:01*	*B*55:01:01 / B*18:01:01*	*C*07:04:01 / C*01:02:01*	*DPB1*01:01:01 / DPB1*02:01:02*	*DQB1*05:03:01 / DQB1*05:02:01*	*DRB1*14:07:01 / DRB1*11:01:01*
CTBD020	*A*24:03:01 / A*24:03:01*	*B*58:01:01 / B*13:01:01*	*C*03:02:02 / C*03:04:01*	*DPB1*02:02:01 / DPB1*04:02:01*	*DQB1*04:02:01 / DQB1*04:02:01*	*DRB1*14:05:01 / DRB1*03:01:01*
CTBD021	*A*02:03:01 / A*24:02:01*	*B*27:04:01 / B*51:01:01*	*C*15:02:01 / C*03:04:01*	*DPB1*02:02:01 / DPB1*02:01:02*	*DQB1*03:01:01 / DQB1*03:01:01*	*DRB1*14:54:01 / DRB1*12:02:01*
CTBD022	*A*26:01:01 / A*02:06:01*	*B*46:01:01 / B*40:01:02*	*C*01:02:01 / C*03:04:01*	*DPB1*13:01:01 / DPB1*13:01:01*	*DQB1*06:01:01 / DQB1*05:02:01*	*DRB1*14:54:01 / DRB1*08:03:02*
CTBD023	*A*11:01:01 / A*11:02:01*	*B*27:04:01 / B*46:01:01*	*C*12:02:02 / C*01:02:01*	*DPB1*02:02:01 / DPB1*47:01:01*	*DQB1*03:01:01 / DQB1*03:01:01*	*DRB1*14:54:01 / DRB1*12:02:01*
CTBD024	*A*24:07:01 / A*24:02:01*	*B*44:03:02 / B*40:01:02*	*C*07:06:01 / C*04:01:01*	*DPB1*04:02:01 / DPB1*03:01:01*	*DQB1*03:01:01 / DQB1*02:02:01*	*DRB1*11:01:01 / DRB1*07:01:01*
CTBD025	*A*02:07:01 / A*11:01:01*	*B*46:01:01 / B*52:01:01*	*C*07:02:01 / C*01:02:01*	*DPB1*02:02:01 / DPB1*13:01:01*	*DQB1*04:02:01 / DQB1*03:03:02*	*DRB1*14:02:01 / DRB1*09:01:02*
CTBD026	*A*33:03:01 / A*24:02:01*	*B*15:02:01 / B*07:02:01*	*C*08:01:01 / C*12:03:01*	*DPB1*04:01:01 / DPB1*04:01:01*	*DQB1*05:01:01 / DQB1*03:01:01*	*DRB1*14:54:01 / DRB1*10:01:01*
CTBD027	*A*24:02:01 / A*11:01:01*	*B*15:25:01 / B*39:01:01*	*C*04:03:01 / C*07:02:01*	*DPB1*05:01:01 / DPB1*05:01:01*	*DQB1*05:02:01 / DQB1*03:01:01*	*DRB1*13:07:01 / DRB1*14:54:01*
CTBD028	*A*02:03:01 / A*11:01:01*	*B*15:18:01 / B*55:01:01*	*C*07:04:01 / C*12:03:01*	*DPB1*02:02:01 / DPB1*13:01:01*	*DQB1*05:01:24 / DQB1*03:01:01*	*DRB1*13:07:01 / DRB1*11:01:01*
CTBD029	*A*02:03:01 / A*02:03:01*	*B*38:02:01 / B*38:02:01*	*C*07:02:01 / C*07:02:01*	*DPB1*13:01:01 / DPB1*05:01:01*	*DQB1*05:01:24 / DQB1*05:02:01*	*DRB1*11:01:01 / DRB1*15:02:01*
CTBD031	*A*32:01:01 / A*11:01:01*	*B*54:01:01 / B*27:05:02*	*C*01:02:01 / C*02:02:02*	*DPB1*09:01:01 / DPB1*05:01:01*	*DQB1*05:01:01 / DQB1*03:01:01*	*DRB1*14:54:01 / DRB1*01:01:01*
CTBD032	*A*24:02:01 / A*11:01:01*	*B*15:12:02 / B*15:01:01*	*C*03:03:01 / C*07:02:01*	*DPB1*04:02:01 / DPB1*13:01:01*	*DQB1*03:01:01 / DQB1*03:01:01*	*DRB1*14:54:01 / DRB1*12:01:01*
CTBD033	*A*24:02:01 / A*11:01:01*	*B*38:02:01 / B*40:01:02*	*C*07:02:01 / C*07:02:01*	*DPB1*04:02:01 / DPB1*13:01:01*	*DQB1*05:01:01 / DQB1*02:02:01*	*DRB1*11:01:01 / DRB1*07:01:01*
CTBD034	*A*02:03:01 / A*02:03:01*	*B*27:04:01 / B*15:25:01*	*C*04:03:01 / C*03:04:01*	*DPB1*04:01:01 / DPB1*05:01:01*	*DQB1*05:02:01 / DQB1*05:02:01*	*DRB1*14:54:01 / DRB1*12:02:01*
CTBD035	*A*24:10:01 / A*11:01:01*	*B*48:01:01 / B*51:01:01*	*C*08:01:01 / C*14:02:01*	*DPB1*01:01:01 / DPB1*39:01:01*	*DQB1*05:02:01 / DQB1*05:02:01*	*DRB1*12:02:01 / DRB1*11:01:01*
CTBD036	*A*02:07:01 / A*30:01:01*	*B*13:02:01 / B*46:01:01*	*C*01:02:01 / C*06:02:01*	*DPB1*17:01:01 / DPB1*04:02:01*	*DQB1*05:03:01 / DQB1*02:02:01*	*DRB1*14:05:01 / DRB1*07:01:01*
CTBD037	*A*11:01:01 / A*11:01:01*	*B*15:02:01 / B*51:01:01*	*C*08:01:01 / C*15:02:01*	*DPB1*04:02:01 / DPB1*13:01:01*	*DQB1*03:01:01 /DQB1*04:02:01*	*DRB1*14:54:01 / DRB1*12:02:01*
CTBD038	*A*24:02:01 / A*11:01:01*	*B*40:01:02 / B*40:01:02*	*C*07:02:01 / C*03:04:01*	*DPB1*09:01:01 / DPB1*02:01:02*	*DQB1*03:03:02 / DQB1*03:02:01*	*DRB1*03:02:01 / DRB1*14:06:01*
CTBD039	*A*26:01:01 / A*11:01:01*	*B*40:01:02 / B*39:01:01*	*C*03:04:01 / C*07:02:99*	*DPB1*05:01:01 / DPB1*13:01:01*	*DQB1*03:01:01 / DQB1*05:02:01*	*DRB1*14:54:01 / DRB1*11:01:01*
CTBD040	*A*24:07:01 / A*26:01:01*	*B*35:05:01 / B*39:01:01*	*C*07:02:01 / C*04:01:01*	*DPB1*02:01:02 / DPB1*04:01:01*	*DQB1*06:01:01 / DQB1*03:01:01*	*DRB1*11:01:01 / DRB1*08:03:02*
CTBD041	*A*02:07:01 / A*24:10:01*	*B*15:21:01 / B*18:01:01*	*C*04:03:01 / C*07:04:01*	*DPB1*13:01:01 / DPB1*04:01:01*	*DQB1*06:01:01 / DQB1*03:01:01*	*DRB1*14:54:01 / DRB1*11:01:01*
CTBD042	*A*33:03:01 / A*11:01:01*	*B*46:01:01 / B*44:03:02*	*C*07:06:01 / C*01:02:01*	*DPB1*05:01:01 / DPB1*03:01:01*	*DQB1*03:01:01 / DQB1*02:02:01*	*DRB1*13:07:01 / DRB1*07:01:01*
CTBD043	*A*02:07:01 / A*24:02:40*	*B*27:04:01 / B*46:01:01*	*C*01:02:01 / C*03:04:01*	*DPB1*10:01:01 / DPB1*02:02:01*	*DQB1*03:03:02 / DQB1*03:01:01*	*DRB1*14:54:01 / DRB1*12:02:01*
CTBD044	*A*23:01:01 / A*33:03:01*	*B*50:01:01 / B*44:03:02*	*C*07:06:01 / C*06:02:01*	*DPB1*13:01:01 / DPB1*02:01:02*	*DQB1*04:02:01 / DQB1*02:02:01*	*DRB1*03:02:01 / DRB1*14:06:01*
CTBD045	*A*33:03:01 / A*03:08:01*	*B*44:03:02 / B*07:02:01*	*C*07:06:01 / C*07:02:01*	*DPB1*05:01:01 / DPB1*04:01:01*	*DQB1*05:02:01 / DQB1*02:02:01*	*DRB1*11:01:01 / DRB1*07:01:01*
CTBD046	*A*02:03:01 / A*11:01:82*	*B*13:01:01 / B*13:01:01*	*C*12:02:02 / C*03:02:02*	*DPB1*14:01:01 / DPB1*02:01:02*	*DQB1*06:01:01 / DQB1*05:02:01*	*DRB1*11:01:01 / DRB1*15:01:01*
CTBD047	*A*24:02:01 / A*03:01:01*	*B*40:02:01 / B*40:01:02*	*C*15:02:01 / C*14:02:01*	*DPB1*13:01:01 / DPB1*02:01:02*	*DQB1*05:01:24 / DQB1*03:01:01*	*DRB1*11:04:01 / DRB1*14:05:01*
CTBD048	*A*24:02:01 / A*03:01:01*	*B*15:12:02 / B*07:02:01*	*C*03:03:01 / C*07:02:01*	*DPB1*02:02:01 / DPB1*02:01:02*	*DQB1*05:01:01 / DQB1*05:02:01*	*DRB1*10:01:01 / DRB1*11:03:01*
CTBD049	*A*11:01:01 / A*11:02:01*	*B*13:01:01 / B*15:02:01*	*C*08:01:01 / C*03:04:01*	*DPB1*14:01:01 / DPB1*03:01:01*	*DQB1*05:01:24 / DQB1*03:01:01*	*DRB1*14:54:01 / DRB1*15:02:01*
CTBD050	*A*33:03:01 / A*02:06:01*	*B*46:01:01 / B*15:02:01*	*C*03:02:02 / C*01:02:01*	*DPB1*02:02:01 / DPB1*04:01:01*	*DQB1*03:03:02 / DQB1*05:02:01*	*DRB1*09:01:02 / DRB1*16:02:01*
CTBD051	*A*24:07:01 / A*68:01:01*	*B*38:02:01 / B*15:02:01*	*C*08:01:01 / C*07:02:01*	*DPB1*02:01:02 / DPB1*04:01:01*	*DPB1*04:01:01 / DQB1*03:01:01*	*DRB1*14:54:01 / DRB1*12:02:01*
CTBD052	*A*02:07:01 / A*11:01:01*	*B*46:01:01 / B*15:02:01*	*C*08:01:01 / C*01:02:01*	*DPB1*13:01:01 / DPB1*03:01:01*	*DQB1*03:01:01 / DQB1*05:01:24*	*DRB1*14:54:01 / DRB1*15:02:01*
CTBD053	*A*11:01:01 / A*01:01:01*	*B*44:03:02 / B*57:01:01*	*C*07:06:01 / C*06:02:01*	*DPB1*04:02:01 / DPB1*02:01:02*	*DQB1*03:03:02 / DQB1*02:02:01*	*DRB1*03:02:01 / DRB1*07:01:01*
CTBD054	*A*02:17:02 / A*24:07:01*	*B*48:01:01 / B*35:05:01*	*C*04:01:01 / C*03:04:01*	*DPB1*02:01:02 / DPB1*05:01:01*	*DQB1*03:01:01 / DQB1*05:02:01*	*DRB1*14:54:01 / DRB1*12:02:01*
CTBD055	*A*11:02:01 / A*02:01:01*	*B*27:04:01 / B*54:01:01*	*C*12:02:02 / C*01:02:01*	*DPB1*02:02:01 / DPB1*05:01:01*	*DQB1*03:01:01 / DQB1*05:02:01*	*DRB1*14:54:01 / DRB1*12:02:01*
CTBD056	*A*01:01:01 / A*11:01:01*	*B*57:01:01 / B*56:01:01*	*C*01:02:01 / C*06:02:01*	*DPB1*31:01:01 / DPB1*13:01:01*	*DQB1*05:01:24 / DQB1*03:01:01*	*DRB1*14:54:01 / DRB1*15:02:01*
CTBD057	*A*02:03:01 / A*11:01:01*	*B*46:01:01 / B*51:02:02*	*C*01:02:01 / C*14:02:01*	*DPB1*05:01:01 / DPB1*03:01:01*	*DQB1*05:01:24 / DQB1*03:03:02*	*DRB1*11:01:01 / DRB1*15:02:01*
CTBD058	*A*33:03:01 / A*11:01:01*	*B*18:01:01 / B*58:01:01*	*C*07:04:01 / C*03:02:02*	*DPB1*02:01:02 / DPB1*13:01:01*	*DQB1*06:09:01 / DQB1*04:02:01*	*DRB1*14:54:01 / DRB1*13:02:01*
CTBD059	*A*11:01:01 / A*11:01:01*	*B*38:02:01 / B*51:01:02*	*C*07:02:01 / C*14:02:01*	*DPB1*02:02:01 / DPB1*14:01:01*	*DQB1*05:03:01 / DQB1*05:02:01*	*DRB1*14:54:01 / DRB1*11:01:01*
CTBD060	*A*02:07:01 / A*33:03:01*	*B*46:01:01 / B*44:03:02*	*C*07:06:01 / C*01:02:01*	*DPB1*14:01:01 / DPB1*02:01:02*	*DQB1*03:03:02 / DQB1*02:02:01*	*DRB1*14:02:01 / DRB1*07:01:01*
CTBD061	*A*33:03:01 / A*24:02:01*	*B*58:01:01 / B*40:01:02*	*C*03:02:02 / C*04:01:01*	*DPB1*05:01:01 / DPB1*04:01:01*	*DQB1*03:01:01 / DQB1*02:01:01*	*DRB1*14:54:01 / DRB1*03:01:01*
CTBD062	*A*02:03:01 / A*01:01:01*	*B*13:01:01 / B*37:01:01*	*C*04:03:01 / C*06:02:01*	*DPB1*03:01:01 / DPB1*02:01:02*	*DQB1*05:01:01 / DQB1*05:01:24*	*DRB1*10:01:01 / DRB1*11:01:01*
CTBD063	*A*74:02:01 / A*24:02:01*	*B*15:02:01 / B*15:15:01*	*C*08:01:01 / C*03:04:01*	*DPB1*19:01:01 / DPB1*03:01:01*	*DQB1*06:02:01 / DQB1*03:01:01*	*DRB1*11:01:01 / DRB1*12:02:01*
CTBD064	*A*33:03:01 / A*11:01:01*	*B*15:01:01 / B*51:01:01*	*C*04:01:01 / C*14:02:01*	*DPB1*05:01:01 / DPB1*05:01:01*	*DQB1*03:01:01 / DQB1*03:01:01*	*DRB1*14:54:01 / DRB1*11:01:01*
CTBD065	*A*24:10:01 / A*68:01:02*	*B*18:01:01 / B*18:01:01*	*C*07:04:01 / C*07:04:01*	*DPB1*13:01:01 / DPB1*13:01:01*	*DQB1*05:01:24 / DQB1*05:01:24*	*DRB1*15:02:01 / DRB1*11:01:01*
CTBD066	*A*31:01:02 / A*24:02:01*	*B*15:02:01 / B*15:11:01*	*C*03:03:01 / C*07:02:01*	*DPB1*21:01 / DPB1*02:01:02*	*DQB1*06:01:01 / DQB1*03:01:01*	*DRB1*14:54:01 / DRB1*08:03:02*
CTBD067	*A*02:07:01 / A*02:07:01*	*B*46:01:01 / B*18:01:01*	*C*01:02:01 / C*07:01:01*	*DPB1*13:01:01 / DPB1*14:01:01*	*DQB1*05:01:24 / DQB1*03:03:02*	*DRB1*11:01:01 / DRB1*15:02:01*
CTBD068	*A*33:03:01 / A*11:01:01*	*B*58:01:01 / B*40:01:02*	*C*03:02:02 / C*07:02:01*	*DPB1*02:02:01 / DPB1*03:01:01*	*DQB1*05:02:01 / DQB1*03:02:01*	*DRB1*11:01:01 / DRB1*15:01:01*
CTBD069	*A*24:02:01 / A*31:01:02*	*B*27:04:01 / B*38:02:01*	*C*07:02:01 / C*03:04:01*	*DPB1*13:01:01 / DPB1*05:01:01*	*DQB1*05:03:01 / DQB1*03:01:01*	*DRB1*14:54:01 / DRB1*12:01:01*
CTBD070	*A*02:03:01 /A*11:01:01*	*B*15:32:01 / B*40:01:02*	*C*07:02:01 / C*12:03:01*	*DPB1*14:01:01 / DPB1*05:01:01*	*DQB1*05:01:24 / DQB1*05:02:01*	*DRB1*14:54:01 / DRB1*12:02:01*
CTBD071	*A*02:07:01 / A*33:03:01*	*B*44:03:02 / B*13:01:01*	*C*07:06:01 / C*03:04:01*	*DPB1*10:01:01 / DPB1*02:01:02*	*DQB1*06:01:01 / DQB1*05:01:01*	*DRB1*11:01:01 / DRB1*10:01:01*
CTBD072	*A*33:03:01 / A*11:01:01*	*B*58:01:01 / B*40:02:01*	*C*03:02:02 / C*15:02:01*	*DPB1*04:01:01 / DPB1*02:01:02*	*DQB1*02:01:01 / DQB1*02:01:01*	*DRB1*03:01:01 / DRB1*14:05:01*
CTBD073	*A*24:03:01 / A*11:01:01*	*B*48:01:01 / B*15:02:01*	*C*08:01:01 / C*08:22:01*	*DPB1*02:01:02 / DPB1*04:01:01*	*DQB1*03:01:01 / DQB1*03:01:01*	*DRB1*14:54:01 / DRB1*12:02:01*
CTBD074	*A*24:02:01 / A*68:01:01*	*B*15:02:01 / B*18:01:01*	*C*08:01:01 / C*07:04:01*	*DPB1*05:01:01 / DPB1*05:01:01*	*DQB1*05:01:24 / DQB1*03:01:01*	*DRB1*14:54:01 / DRB1*11:03:01*
CTBD075	*A*02:07:01 / A*03:01:01*	*B*46:01:01 / B*35:03:01*	*C*01:02:01 / C*12:03:01*	*DPB1*17:01:01 / DPB1*05:01:01*	*DQB1*03:03:02 / DQB1*02:02:01*	*DRB1*07:01:01 DRB1*14:02:01*
CTBD076	*A*02:03:01 / A*24:02:01*	*B*38:02:01 / B*37:01:01*	*C*07:02:01 / C*06:02:01*	*DPB1*09:01:01 / DPB1*13:01:01*	*DQB1*05:01:01 / DQB1*06:01:01*	*DRB1*10:01:01 / DRB1*08:03:02*
CTBD077	*A*24:02:01 / A*11:01:01*	*B*55:02:01 / B*35:01:01*	*C*04:03:01 / C*01:02:01*	*DPB1*02:01:02 / DPB1*05:01:01*	*DQB1*05:03:01 / DQB1*03:02:01*	*DRB1*14:54:01 / DRB1*03:02:01*
CTBD078	*A*02:07:01 / A*30:01:01*	*B*13:02:01 / B*46:01:01*	*C*01:02:01 / C*06:02:01*	*DPB1*17:01:01 / DPB1*14:01:01*	*DQB1*06:01:01 / DQB1*02:02:01*	*DRB1*08:03:02 / DRB1*07:01:01*
CTBD079	*A*24:02:40 / A*11:01:01*	*B*15:02:01 / B*15:02:01*	*C*08:01:01 / C*08:22:01*	*DPB1*02:01:02 / DPB1*04:02:01*	*DQB1*05:02:01 / DQB1*03:01:01*	*DRB1*14:54:01 / DRB1*12:02:01*
CTBD080	*A*02:07:01 / A*11:01:01*	*B*46:01:01 / B*40:01:02*	*C*07:02:01 / C*01:02:01*	*DPB1*02:01:02 / DPB1*05:01:01*	*DQB1*06:01:01 / DQB1*03:01:01*	*DRB1*11:01:01 / DRB1*08:03:02*
CTBD081	*A*02:07:01 / A*30:01:01*	*B*13:02:01 / B*35:05:01*	*C*06:02:01 / C*18:02:01*	*DPB1*17:01:01 / DPB1*17:01:01*	*DQB1*03:01:01 / DQB1*02:02:01*	*DRB1*14:54:01 / DRB1*07:01:01*
CTBD082	*A*11:01:01 / A*11:01:01*	*B*13:01:01 / B*15:02:01*	*C*04:06:01 / C*08:01:01*	*DPB1*26:01:02 / DPB1*13:01:01*	*DQB1*03:03:02 / DQB1*05:02:01*	*DRB1*14:54:01 / DRB1*12:02:01*
CTBD083	*A*24:07:01 / A*24:20:01*	*B*15:02:01 / B*55:01:01*	*C*08:01:01 / C*12:03:01*	*DPB1*02:01:02 / DPB1*02:01:02*	*DQB1*06:01:01 / DQB1*05:03:01*	*DRB1*11:01:01 / DRB1*14:54:01*
CTBD084	*A*02:03:01 / A*11:01:01*	*B*15:25:01 / B*52:01:01*	*C*04:03:01 / C*12:02:02*	*DPB1*05:01:01 / DPB1*04:01:01*	*DQB1*06:01:01 / DQB1*05:02:01*	*DRB1*11:01:01 / DRB1*12:02:01*
CTBD085	*A*74:02:01 / A*24:02:01*	*B*58:01:01 / B*35:05:01*	*C*01:02:01 / C*04:01:01*	*DPB1*13:01:01 / DPB1*04:01:01*	*DQB1*05:01:24 / DQB1*05:01:24*	*DRB1*03:01:01 / DRB1*03:01:01*

Novel Genetic Variants of HLA Gene Associated with Thai Behcet's Disease (BD) Patients Using Next Generation Sequencing Technology.

Supplement 1 : HLA genotyping of Behcet's and Control

TypeN0	HLA-A	HLA-B	HLA-C	HLA-DPB1	HLA-DQB11	HLA-RB1
CTBD086	*A*02:03:01 / A*11:01:01*	*B*38:02:01 / B*13:01:01*	*C*04:06:01 / C*07:02:01*	*DPB1*31:01:01 / DPB1*05:01:01*	*DQB1*03:03:02 / DQB1*03:01:01*	*DRB1*14:54:01 / DRB1*12:02:01*
CTBD087	*A*02:03:01 / A*33:03:01*	*B*44:03:02 / B*38:02:01*	*C*07:06:01 / C*07:02:01*	*DPB1*05:01:01 / DPB1*04:02:01*	*DQB1*05:02:01 / DQB1*03:02:01*	*DRB1*11:01:01 / DRB1*16:02:01*
CTBD088	*A*02:07:01 / A*24:02:01*	*B*46:01:01 / B*51:01:01*	*C*15:02:01 / C*01:02:01*	*DPB1*05:01:01 / DPB1*02:01:02*	*DQB1*03:03:02 / DQB1*05:02:01*	*DRB1*14:54:01 / DRB1*09:01:02*
CTBD089	*A*24:02:01 / A*11:01:01*	*B*38:02:01 / B*51:02:01*	*C*15:02:01 / C*07:02:01*	*DPB1*13:01:01 / DPB1*02:01:02*	*DQB1*05:01:24 / DQB1*05:01:24*	*DRB1*11:01:01 / DRB1*15:02:01*
CTBD090	*A*24:02:01 / A*11:01:01*	*B*15:02:01 / B*46:01:01*	*C*08:01:01 / C*08:22:01*	*DPB1*09:01:01 / DPB1*03:01:01*	*DQB1*06:01:01 / DQB1*03:01:01*	*DRB1*14:54:01 / DRB1*11:01:01*
CTBD091	*A*11:01:01 / A*11:01:01*	*B*40:01:02 / B*40:01:02*	*C*08:01:01 / C*07:02:01*	*DPB1*14:01:01 / DPB1*10:01:01*	*DQB1*03:01:01 / DQB1*03:01:01*	*DRB1*14:54:01 / DRB1*12:02:01*
CTBD092	*A*26:01:01 / A*11:01:01*	*B*52:01:01 / B*38:02:01*	*C*07:02:01 / C*07:02:01*	*DPB1*05:01:01 / DPB1*22:01:01*	*DQB1*03:01:01 / DQB1*05:02:01*	*DRB1*14:54:01 / DRB1*12:02:01*
CTBD093	*A*33:03:01 A*11:01:01*	*B*44:03:02 / B*51:01:01*	*C*07:06:01 / C*14:02:01*	*DPB1*02:01:02 / DPB1*04:02:01*	*DQB1*03:03:02 / DQB1*02:02:01*	*DRB1*07:01:01 / DRB1*03:02:01*
CTBD094	*A*02:07:01 / A*24:02:01*	*B*27:04:01 / B*46:01:01*	*C*01:02:01 / C*04:01:01*	*DPB1*04:01:01 / DPB1*05:01:01*	*DQB1*03:01:01 / DQB1*05:02:01*	*DRB1*14:54:01 / DRB1*11:01:01*
CTBD095	*A*24:07:01 / A*33:03:01*	*B*15:02:01 / B*58:01:01*	*C*08:01:01 / C*03:02:02*	*DPB1*04:01:01 / DPB1*05:01:01*	*DQB1*05:01:24 /DQB1*05:01:24*	*DRB1*03:01:01 / DRB1*11:01:01*
CTBD096	*A*02:07:01 / A*11:02:01*	*B*46:01:01 / B*15:02:01*	*C*08:01:01 / C*01:02:01*	*DPB1*02:02:01 / DPB1*13:01:01*	*DQB1*06:01:01 / DQB1*03:03:02*	*DRB1*08:03:02 / DRB1*09:01:02*
CTBD097	*A*11:01:01 / A*33:03:01*	*B*44:03:02 / B*15:18:01*	*C*07:06:01 / C*08:01:01*	*DPB1*05:01:01 / DPB1*04:01:01*	*DQB1*03:03:02 / DQB1*03:02:01*	*DRB1*03:02:01 / DRB1*09:01:02*
CTBD098	*A*02:03:01 / A*11:01:01*	*B*15:02:01 / B*40:01:02*	*C*08:01:01 / C*07:02:01*	*DPB1*02:01:02 / DPB1*47:01:01*	*DQB1*04:02:01 / DQB1*03:01:01*	*DRB1*14:54:01 / DRB1*12:02:01*
CTBD099	*A*33:03:01 / A*03:01:01*	*B*58:01:01 / B*35:01:01*	*C*03:02:02 / C*12:03:01*	*DPB1*04:01:01 / DPB1*03:01:01*	*DQB1*05:01:01 / DQB1*06:03:01*	*DRB1*14:05:01 / DRB1*10:01:01*
CTBD100	*A*26:01:01 / A*03:01:01*	*B*35:05:01 / B*15:01:01*	*C*04:01:01 / C*04:01:01*	*DPB1*05:01:01 / DPB1*13:01:01*	*DQB1*03:01:01 / DQB1*03:02:01*	*DRB1*14:54:01 / DRB1*12:02:01*
CTBD101	*A*24:02:01 / A*02:01:01*	*B*15:11:01 / B*15:01:01*	*C*03:03:01 / C*04:01:01*	*DPB1*02:02:01 / DPB1*05:01:01*	*DQB1*06:01:01 / DQB1*03:02:01*	*DRB1*08:03:02 / DRB1*03:02:01*
CTBD102	*A*02:07:01 / A*31:01:02*	*B*46:01:01 / B*51:01:01*	*C*15:02:01 / C*01:02:01*	*DPB1*05:01:01 / DPB1*05:01:01*	*DQB1*05:02:01 / DQB1*05:02:01*	*DRB1*16:02:01 / DRB1*11:01:01*
CTBD103	*A*24:02:01 / A*11:01:01*	*B*07:02:01 / B*15:01:01*	*C*07:02:01 / C*04:01:01*	*DPB1*02:02:01 / DPB1*05:01:01*	*DQB1*03:02:01 / DQB1*03:01:01*	*DRB1*14:05:01 / DRB1*11:04:01*
CTBD104	*A*74:02:01 / A*11:01:01*	*B*15:02:01 / B*40:01:02*	*C*08:01:01 / C*04:01:01*	*DPB1*13:01:01 / DPB1*04:01:01*	*DQB1*03:01:01 / DQB1*05:02:01*	*DRB1*11:01:01 / DRB1*14:54:01*
CTBD105	*A*11:01:01 / A*11:01:01*	*B*40:01:02 / B*40:02:01*	*C*15:02:01 / C*07:02:01*	*DPB1*13:01:01 / DPB1*05:01:01*	*DQB1*03:03:02 / DQB1*05:02:01*	*DRB1*14:54:01 / DRB1*12:02:01*
CTBD106	*A*02:07:01 / A*02:01:01*	*B*67:01:01 / B*18:01:01*	*C*07:02:01 / C*07:02:01*	*DPB1*02:01:02 / DPB1*02:02:01*	*DQB1*05:02:01 / DQB1*03:02:01*	*DRB1*03:02:01 / DRB1*16:02:01*
CTBD107	*A*11:01:01 / A*11:01:01*	*B*46:01:01/ B*15:02:01*	*C*08:01:01 / C*01:02:01*	*DPB1*21:01 / DPB1*26:01:02*	*DQB1*03:01:01 / DQB1*05:02:01*	*DRB1*14:54:01 / DRB1*12:02:01*
CTBD108	*A*24:07:01 / A*33:03:01*	*B*35:05:01 / B*44:03:02*	*C*07:06:01 / C*04:01:01*	*DPB1*03:01:01 / DPB1*04:01:01*	*DQB1*05:01:24 / DQB1*02:02:01*	*DRB1*11:01:01 / DRB1*07:01:01*
CTBD109	*A*24:02:01 / A*24:02:01*	*B*13:01:01 / B*35:03:01*	*C*07:06:01 / C*03:04:01*	*DPB1*04:01:01 / DPB1*04:01:01*	*DQB1*03:01:01 / DQB1*05:02:01*	*DRB1*11:01:01 / DRB1*14:05:01*
CTBD110	*A*02:03:01 /A*02:07:01*	*B*46:01:01 / B*46:01:01*	*C*01:02:01 / C*01:02:01*	*DPB1*17:01:01 / DPB1*05:01:01*	*DQB1*03:03:02 / DQB1*02:02:01*	*DRB1*03:02:01 / DRB1*14:02:01*
CTBD111	*A*30:01:01 / A*11:01:01*	*B*13:02:01 / B*52:01:01*	*C*12:02:02 / C*06:02:01*	*DPB1*02:01:02 / DPB1*02:01:02*	*DQB1*03:02:01 / DQB1*03:02:01*	*DRB1*03:02:01 / DRB1*04:03:01*
CTBD112	*A*30:01:01 / A*24:02:01*	*B*13:02:01 / B*40:01:02*	*C*07:02:01 / C*06:02:01*	*DPB1*17:01:01 / DPB1*05:01:01*	*DQB1*04:02:01 / DQB1*02:02:01*	*DRB1*03:02:01 / DRB1*14:02:01*
CTBD113	*A*11:01:01 / A*11:01:01*	*B*13:01:01 / B*51:02:01*	*C*15:02:01 / C*03:04:01*	*DPB1*04:01:01 / DPB1*05:01:01*	*DQB1*06:02:01 / DQB1*05:02:01*	*DRB1*11:01:01 / DRB1*15:01:01*
CTBD114	*A*33:03:01 / A*01:01:01*	*B*44:03:02 / B*37:01:01*	*C*07:06:01 / C*06:02:01*	*DPB1*09:01:01 / DPB1*04:02:01*	*DQB1*05:01:01 / DQB1*02:02:01*	*DRB1*10:01:01 / DRB1*07:01:01*
CTBD115	*A*02:03:01 / A*11:01:01*	*B*46:01:01 / B*40:01:02*	*C*01:02:01 / C*03:03:01*	*DPB1*13:01:01 / DPB1*05:01:01*	*DQB1*05:01:24 / DQB1*04:02:01*	*DRB1*15:02:01 / DRB1*04:05:01*
CTBD116	*A*33:03:01 / A*03:01:01*	*B*44:03:02 / B*07:02:01*	*C*07:06:01 / C*07:02:01*	*DPB1*04:01:01 / DPB1*04:01:01*	*DQB1*05:01:01 / DQB1*02:02:01*	*DRB1*07:01:01 / DRB1*10:01:01*
CTBD117	*A*11:01:01 / A*11:02:01*	*B*15:02:01 / B*27:04:01*	*C*08:01:01 / C*12:02:02*	*DPB1*02:01:02 / DPB1*02:02:01*	*DQB1*03:01:01 / DQB1*03:02:01*	*DRB1*14:54:01 / DRB1*12:02:01*
CTBD118	*A*02:07:01 / A*33:03:01*	*B*46:01:01 / B*44:03:02*	*C*07:06:01 / C*01:02:01*	*DPB1*05:01:01 / DPB1*04:01:01*	*DQB1*05:02:01 / DQB1*02:02:01*	*DRB1*16:02:01 / DRB1*07:01:01*
CTBD119	*A*24:07:01 / A*02:01:01*	*B*27:04:01 / B*13:01:01*	*C*04:06:01 / C*03:04:01*	*DPB1*13:01:01 / DPB1*04:02:01*	*DQB1*06:01:01 / DQB1*05:01:24*	*DRB1*15:02:01 / DRB1*08:03:02*
CTBD120	*A*30:01:01 / A*11:01:01*	*B*13:02:01 / B*15:02:01*	*C*08:01:01 / C*06:02:01*	*DPB1*05:01:01 / DPB1*04:02:01*	*DQB1*03:01:01 / DQB1*03:02:01*	*DRB1*11:03:01 / DRB1*14:05:01*
CTBD121	*A*02:03:01 / A*11:01:01*	*B*27:04:01 / B*39:09:01*	*C*12:02:02 / C*07:02:01*	*DPB1*05:01:01 / DPB1*03:01:01*	*DQB1*03:01:01 / DQB1*05:03:01*	*DRB1*14:05:01 / DRB1*12:02:01*
CTBD122	*A*02:07:01 / A*11:02:01*	*B*38:02:01 / B*40:01:02*	*C*07:02:01 / C*07:02:01*	*DPB1*02:01:02 / DPB1*47:01:01*	*DQB1*05:02:01 / DQB1*05:02:01*	*DRB1*11:01:01 / DRB1*15:02:01*
CTBD123	*A*02:07:01 / A*33:03:01*	*B*46:01:01 / B*58:01:01*	*C*03:02:02 / C*01:02:01*	*DPB1*31:01:01 / DPB1*04:01:01*	*DQB1*05:03:01 / DQB1*05:03:01*	*DRB1*14:05:01 / DRB1*03:01:01*
CTBD124	*A*24:02:01 / A*11:01:01*	*B*46:01:01 / B*18:01:01*	*C*07:04:01 / C*01:02:01*	*DPB1*04:01:01 / DPB1*02:01:02*	*DQB1*03:01:01 / DQB1*05:02:01*	*DRB1*14:54:01 / DRB1*12:02:01*
CTBD125	*A*33:01:01 / A*01:01:01*	*B*37:01:01 / B*52:01:01*	*C*07:02:01 / C*06:02:01*	*DPB1*04:02:01 / DPB1*04:01:01*	*DQB1*05:01:01 / DQB1*03:02:01*	*DRB1*10:01:01 / DRB1*04:05:01*
CTBD126	*A*02:07:01 / A*02:01:01*	*B*46:01:01 / B*52:01:01*	*C*12:02:02 / C*01:02:01*	*DPB1*21:01 / DPB1*04:01:01*	*DQB1*05:02:01 / DQB1*02:02:01*	*DRB1*14:54:01 / DRB1*07:01:01*
CTBD127	*A*02:07:01 / A*02:01:01*	*B*46:01:01 / B*15:02:01*	*C*08:01:01 / C*01:02:01*	*DPB1*05:01:01 / DPB1*05:01:01*	*DQB1*03:03:02 / DQB1*05:02:01*	*DRB1*11:01:01 / DRB1*16:02:01*
CTBD128	*A*33:03:01 / A*11:01:01*	*B*58:01:01 / B*15:01:01*	*C*03:02:02 / C*04:01:01*	*DPB1*02:01:02 / DPB1*02:01:02*	*DQB1*02:01:01 / DQB1*03:02:01*	*DRB1*14:05:01 / DRB1*03:01:01*
CTBD129	*A*11:01:01 / A*11:01:01*	*B*51:01:02 / B*51:01:02*	*C*14:02:01 / C*14:02:01*	*DPB1*02:02:01 / DPB1*02:01:02*	*DQB1*05:03:01 / DQB1*05:03:01*	*DRB1*14:54:01 / DRB1*14:05:01*
CTBD130	*A*02:07:01 / A*33:03:01*	*B*58:01:01 / B*46:01:01*	*C*03:02:02 / C*01:02:01*	*DPB1*09:01:01 / DPB1*05:01:01*	*DQB1*06:09:01 / DQB1*05:02:01*	*DRB1*13:02:01 / DRB1*14:54:01*
CTBD131	*A*33:03:01 / A*24:07:01*	*B*58:01:01 / B*40:02:01*	*C*15:02:01 / C*03:02:02*	*DPB1*05:01:01 / DPB1*04:01:01*	*DQB1*06:09:01 / DQB1*03:03:02*	*DRB1*14:54:01 / DRB1*13:02:01*
CTBD132	*A*11:01:01 / A*11:01:01*	*B*27:04:01 / B*37:01:01*	*C*03:04:01 / C*06:02:01*	*DPB1*10:01:01 / DPB1*03:01:01*	*DQB1*05:01:01 / DQB1*05:02:01*	*DRB1*10:01:01 / DRB1*11:01:01*
CTBD133	*A*02:07:01 / A*24:02:01*	*B*54:01:01 / B*46:01:01*	*C*01:02:01 / C*07:06:01*	*DPB1*22:01:01 / DPB1*05:01:01*	*DQB1*06:01:01 / DQB1*03:03:02*	*DRB1*08:03:02 / DRB1*09:01:02*
CTBD134	*A*02:07:01 / A*02:01:01*	*B*15:02:01 / B*40:01:02*	*C*08:01:01 / C*07:02:01*	*DPB1*05:01:01 / DPB1*03:01:01*	*DQB1*05:02:01 / DQB1*02:02:01*	*DRB1*14:54:01 / DRB1*07:01:01*
CTBD135	*A*02:03:01 / A*11:01:01*	*B*46:01:01 / B*13:01:01*	*C*03:04:01 / C*01:02:01*	*DPB1*13:01:01 / DPB1*13:01:01*	*DQB1*05:02:01 / DQB1*05:02:01*	*DRB1*14:54:01 / DRB1*11:01:01*
CTBD136	*A*02:09:01 / A*24:02:01*	*B*44:03:02 / B*40:01:02*	*C*07:06:01 / C*03:03:01*	*DPB1*04:02:01 / DPB1*05:01:01*	*DQB1*04:02:01 / DQB1*02:02:01*	*DRB1*14:02:01 / DRB1*07:01:01*
CTBD138	*A*33:03:01 / A*03:01:01*	*B*58:01:01 / B*07:02:01*	*C*03:02:02 / C*07:02:01*	*DPB1*02:01:02 / DPB1*05:01:01*	*DQB1*06:04:01 / DQB1*02:01:01*	*DRB1*03:01:01 / DRB1*14:54:01*
CTBD139	*A*33:03:01 / A*11:01:01*	*B*40:06:01 / B*15:02:01*	*C*08:01:01 / C*15:02:01*	*DPB1*26:01:02 / DPB1*13:01:01*	*DQB1*03:01:01 / DQB1*05:02:01*	*DRB1*11:01:01 / DRB1*14:05:01*
CTBD140	*A*02:07:01 / A*02:01:01*	*B*40:01:02 / B*40:01:02*	*C*07:02:01 / C*07:02:01*	*DPB1*05:01:01 / DPB1*13:01:01*	*DQB1*03:03:02 / DQB1*03:02:01*	*DRB1*14:02:01 / DRB1*09:01:02*
CTBD141	*A*02:07:01 /A*33:03:01*	*B*46:01:01 / B*44:03:02*	*C*07:06:01 / C*01:02:01*	*DPB1*14:01:01 / DPB1*05:01:01*	*DQB1*05:02:01 / DQB1*02:02:01*	*DRB1*14:54:01 / DRB1*07:01:01*
CTBD142	*A*24:07:01 / A*11:01:01*	*B*15:32:01 / B*15:02:01*	*C*08:01:01 / C*12:03:01*	*DPB1*05:01:01 / DPB1*05:01:01*	*DQB1*06:01:01 / DQB1*06:01:01*	*DRB1*11:01:01 / DRB1*15:01:01*
CTBD143	*A*33:03:01 / A*01:01:01*	*B*57:01:01 / B*44:03:02*	*C*07:06:01 / C*06:02:01*	*DPB1*03:01:01 / DPB1*06:01:01*	*DQB1*03:03:02 / DQB1*02:02:01*	*DRB1*07:01:01 / DRB1*14:02:01*
CTBD144	*A*02:03:01 / A*11:01:01*	*B*07:02:01 / B*18:01:01*	*C*07:04:01 / C*07:02:01*	*DPB1*13:01:01 / DPB1*39:01:01*	*DQB1*03:01:01 / DQB1*05:01:24*	*DRB1*14:54:01 / DRB1*15:02:01*
CTBD145	*A*02:03:01 / A*02:03:01*	*B*38:02:01 / B*38:02:01*	*C*07:02:01 / C*07:02:01*	*DPB1*13:01:01 / DPB1*02:01:02*	*DQB1*05:01:24 / DQB1*03:02:01*	*DRB1*15:02:01 / DRB1*11:01:01*
CTBD146	*A*02:03:01 / A*03:01:01*	*B*07:02:01 / B*18:01:01*	*C*07:04:01 / C*07:02:01*	*DPB1*09:01:01 / DPB1*01:01:01*	*DQB1*05:01:01 / DQB1*05:03:01*	*DRB1*14:54:01 / DRB1*10:01:01*
CTBD147	*A*74:02:01 / A*02:01:01*	*B*48:01:01 / B*51:01:02*	*C*14:02:01 / C*14:02:01*	*DPB1*13:01:01 / DPB1*05:01:01*	*DQB1*05:03:01 / DQB1*03:01:01*	*DRB1*14:05:01 / DRB1*14:54:01*
CTBD148	*A*24:02:01 /A*24:20:01*	*B*27:04:01 / B*40:02:01*	*C*03:03:01 / C*07:02:01*	*DPB1*05:01:01 / DPB1*05:01:01*	*DQB1*03:01:01 / DQB1*03:01:01*	*DRB1*14:54:01 / DRB1*12:02:01*
CTBD149	*A*02:07:01 / A*30:01:01*	*B*13:02:01 / B*46:01:01*	*C*01:02:01 / C*01:02:01*	*DPB1*02:02:01 / DPB1*05:01:01*	*DQB1*03:03:02 / DQB1*05:02:01*	*DRB1*14:54:01 / DRB1*09:01:02*
CTBD150	*A*33:03:01 / A*11:01:01*	*B*58:01:01 / B*51:02:01*	*C*03:02:02 / C*15:02:01*	*DPB1*01:01:01 / DPB1*04:02:01*	*DQB1*05:01:01 / DQB1*03:02:01*	*DRB1*01:01:01 / DRB1*14:02:01*
CTBD151	*A*33:03:01 / A*11:01:01*	*B*40:06:01 / B*15:02:01*	*C*08:01:01 / C*15:02:01*	*DPB1*02:01:02 / DPB1*04:01:01*	*DQB1*03:01:01 / DQB1*03:02:01*	*DRB1*14:54:01 / DRB1*12:02:01*
CTBD152	*A*24:10:01 / A*24:03:01*	*B*13:01:01 / B*18:01:01*	*C*07:04:01 / C*03:04:01*	*DPB1*13:01:01 / DPB1*05:01:01*	*DQB1*05:01:24 / DQB1*03:01:01*	*DRB1*11:01:01 / DRB1*15:02:01*
CTBD153	*A*33:03:01 / A*11:01:01*	*B*15:25:01 / B*58:01:01*	*C*04:03:01 / C*03:02:02*	*DPB1*04:01:01 / DPB1*34:01:01*	*DQB1*06:09:01 / DQB1*06:09:01*	*DRB1*03:01:01 / DRB1*13:02:01*
CTBD154	*A*02:07:01 / A*32:01:01*	*B*15:12:02 / B*27:05:02*	*C*03:03:01 / C*02:02:02*	*DPB1*01:01:01 / DPB1*04:01:01*	*DQB1*03:01:01 / DQB1*06:03:01*	*DRB1*14:54:01 / DRB1*13:01:01*
CTBD155	*A*33:03:01 / A*11:01:01*	*B*46:01:01 / B*44:03:02*	*C*07:06:01 / C*01:02:01*	*DPB1*04:02:01 / DPB1*05:01:01*	*DQB1*03:03:02 / DQB1*03:03:02*	*DRB1*03:02:01 / DRB1*14:02:01*
CTBD156	*A*11:01:01 / A*11:01:01*	*B*15:02:01 / B*51:02:01*	*C*08:01:01 / C*15:02:01*	*DPB1*02:01:02 / DPB1*05:01:01*	*DQB1*03:01:01 / DQB1*03:02:01*	*DRB1*14:54:01 / DRB1*12:02:01*

Novel Genetic Variants of HLA Gene Associated with Thai Behcet’s Disease (BD) Patients Using Next Generation Sequencing Technology.

Supplement 1 : HLA genotyping of Behcet's and Control

TypeN0	HLA-A	HLA-B	HLA-C	HLA-DPB1	HLA-DQB11	HLA-RB1
CTBD157	*A*29:01:01 / A*24:02:01*	*B*07:05:01 / B*52:01:01*	*C*15:05:02 / C*07:02:01*	*DPB1*39:01:01 / DPB1*02:01:02*	*DQB1*05:01:01 / DQB1*03:01:01*	*DRB1*10:01:01 / DRB1*10:01:01*
CTBD158	*A*33:03:01 / A*33:03:01*	*B*58:01:01 / B*53:01:01*	*C*03:02:02 / C*04:01:01*	*DPB1*02:01:02 / DPB1*14:01:01*	*DQB1*05:01:01 / DQB1*05:03:01*	*DRB1*14:05:01 / DRB1*10:01:01*
CTBD159	*A*11:01:01 / A*26:01:01*	*B*46:01:01 / B*55:02:01*	*C*01:02:01 / C*01:02:01*	*DPB1*05:01:01 / DPB1*05:01:01*	*DQB1*04:02:01 / DQB1*03:03:02*	*DRB1*14:02:01 / DRB1*03:02:01*
CTBD160	*A*01:01:01 / A*11:01:01*	*B*57:01:01 / B*07:02:01*	*C*07:02:01 / C*06:02:01*	*DPB1*03:01:01 / DPB1*03:01:01*	*DQB1*03:01:01 / DQB1*05:02:01*	*DRB1*14:54:01 / DRB1*11:03:01*
CTBD161	*A*11:01:01 / A*11:01:01*	*B*15:02:01 / B*07:02:01*	*C*08:01:01 / C*07:02:01*	*DPB1*14:01:01 /DPB1*14:01:01*	*DQB1*03:01:01 / DQB1*05:02:01*	*DRB1*14:54:01 / DRB1*11:01:01*
CTBD162	*A*33:03:01 / A*24:02:01*	*B*58:01:01 / B*15:02:01*	*C*08:01:01 / C*03:02:02*	*DPB1*13:01:01 / DPB1*05:01:01*	*DQB1*04:02:01 / DQB1*02:01:01*	*DRB1*03:01:01 / DRB1*14:05:01*
CTBD163	*A*01:01:01 / A*01:01:01*	*B*57:01:01 / B*51:06:01*	*C*12:03:01 / C*06:02:01*	*DPB1*09:01:01 / DPB1*09:01:01*	*DQB1*03:03:02 / DQB1*03:03:02*	*DRB1*07:01:01 / DRB1*14:02:01*
CTBD164	*A*33:03:01 / A*11:01:01*	*B*40:01:02 / B*51:01:01*	*C*15:02:01 / C*07:02:01*	*DPB1*05:01:01 / DPB1*05:01:01*	*DQB1*06:01:01 / DQB1*05:02:01*	*DRB1*08:03:02 / DRB1*14:54:01*
CTBD165	*A*02:07:01 / A*24:02:01*	*B*46:01:01 / B*35:03:01*	*C*01:02:01 / C*04:01:01*	*DPB1*02:02:01 / DPB1*05:01:01*	*DQB1*03:03:02 / DQB1*03:02:01*	*DRB1*03:02:01 / DRB1*14:06:01*
CTBD166	*A*24:10:01 / A*02:01:01*	*B*40:01:02 / B*18:01:01*	*C*07:04:01 / C*03:04:01*	*DPB1*01:01:01 / DPB1*05:01:01*	*DQB1*04:02:01 / DQB1*05:01:24*	*DRB1*15:02:01 / DRB1*11:01:01*
CTBD167	*A*30:01:01 / A*01:01:01*	*B*57:01:01 / B*13:02:01*	*C*06:02:01 / C*06:02:01*	*DPB1*17:01:01 / DPB1*02:01:02*	*DQB1*03:03:02 / DQB1*02:02:01*	*DRB1*07:01:01 / DRB1*03:02:01*
CTBD168	*A*33:03:01 / A*01:01:01*	*B*44:03:02 / B*37:01:01*	*C*07:06:01 / C*06:02:01*	*DPB1*21:01 / DPB1*02:01:02*	*DQB1*05:01:01 / DQB1*05:02:01*	*DRB1*14:54:01 / DRB1*10:01:01*
CTBD169	*A*02:03:01 / A*24:02:01*	*B*13:01:01 / B*07:02:01*	*C*12:02:02 / C*07:02:01*	*DPB1*13:01:01 / DPB1*05:01:01*	*DQB1*03:03:02 / DQB1*05:03:01*	*DRB1*14:04:01 / DRB1*09:01:02*
CTBD170	*A*29:01:01 / A*11:01:01*	*B*07:05:01 / B*15:02:01*	*C*08:01:01 / C*15:05:02*	*DPB1*02:01:02 / DPB1*13:01:01*	*DQB1*05:01:01 / DQB1*05:02:01*	*DRB1*10:01:01 / DRB1*11:01:01*
CTBD171	*A*02:07:01 / A*24:02:40*	*B*46:01:01 / B*15:01:01*	*C*01:02:01 / C*04:01:01*	*DPB1*05:01:01 / DPB1*05:01:01*	*DQB1*03:03:02 / DQB1*03:03:02*	*DRB1*09:01:02 / DRB1*14:06:01*
CTBD172	*A*24:07:01 / A*11:01:01*	*B*52:01:01 / B*40:02:01*	*C*07:02:01 / C*15:02:01*	*DPB1*01:01:01 / DPB1*01:01:01*	*DQB1*05:03:01 / DQB1*05:02:01*	*DRB1*14:54:01 / DRB1*12:02:01*
CTBD173	*A*11:02:01 / A*26:01:01*	*B*08:01:01 / B*55:01:01*	*C*12:02:02 / C*07:02:01*	*DPB1*05:01:01 / DPB1*03:01:01*	*DQB1*03:03:02 / DQB1*05:03:01*	*DRB1*14:05:01 / DRB1*09:01:02*
CTBD174	*A*30:01:01 / A*30:01:12*	*B*38:02:01 / B*52:01:01*	*C*07:02:01 / C*07:02:01*	*DPB1*10:01:01 / DPB1*13:01:01*	*DQB1*03:01:01 / DQB1*05:01:24*	*DRB1*14:54:01 / DRB1*11:01:01*
CTBD175	*A*33:03:01 / A*11:01:01*	*B*58:01:01 / B*46:01:01*	*C*03:02:02 / C*01:02:01*	*DPB1*02:01:02 / DPB1*03:01:01*	*DQB1*06:01:01 / DQB1*03:02:01*	*DRB1*13:07:01 / DRB1*15:02:01*
CTBD176	*A*02:03:01 / A*33:03:01*	*B*44:03:02 / B*40:01:02*	*C*07:06:01 / C*07:02:01*	*DPB1*03:01:01 / DPB1*03:01:01*	*DQB1*03:01:01 / DQB1*05:01:24*	*DRB1*14:54:01 / DRB1*11:01:01*
CTBD177	*A*33:03:01 / A*24:02:01*	*B*15:12:02 / B*35:03:01*	*C*03:03:01 / C*12:03:01*	*DPB1*03:01:01 / DPB1*05:01:01*	*DQB1*05:02:01 / DQB1*03:01:01*	*DRB1*11:01:01 / DRB1*14:05:01*
CTBD178	*A*02:03:01 / A*02:03:01*	*B*56:01:01 / B*39:09:01*	*C*07:02:01 / C*01:02:01*	*DPB1*13:01:01 / DPB1*02:01:02*	*DQB1*03:02:01 / DQB1*03:01:01*	*DRB1*13:07:01 / DRB1*14:54:01*
CTBD179	*A*11:01:01 / A*02:06:01*	*B*40:06:01 / B*13:01:01*	*C*08:01:01 / C*03:04:01*	*DPB1*13:01:01 / DPB1*05:01:01*	*DQB1*05:01:24 / DQB1*05:02:01*	*DRB1*14:54:01 / DRB1*15:02:01*
CTBD180	*A*24:07:01 / A*02:03:01*	*B*35:05:01 / B*35:05:01*	*C*04:01:01 / C*04:01:01*	*DPB1*04:01:01 / DPB1*02:01:02*	*DQB1*03:01:01 / DQB1*03:01:01*	*DRB1*14:54:01 / DRB1*12:01:01*
CTBD181	*A*02:03:01 / A*33:03:01*	*B*40:06:01 / B*40:01:02*	*C*04:03:01 / C*15:02:01*	*DPB1*14:01:01 / DPB1*03:01:01*	*DQB1*03:01:01 / DQB1*05:02:01*	*DRB1*14:54:01 / DRB1*12:02:01*
CTBD182	*A*11:01:01 / A*02:01:01*	*B*56:01:01 / B*40:01:02*	*C*07:02:01 / C*07:02:01*	*DPB1*02:01:02 / DPB1*05:01:01*	*DQB1*03:03:02 / DQB1*03:02:01*	*DRB1*03:02:01 / DRB1*14:02:01*
CTBD183	*A*02:03:01 / A*24:02:01*	*B*46:01:01 / B*46:01:01*	*C*01:02:01 / C*01:02:01*	*DPB1*26:01:02 / DPB1*02:02:01*	*DQB1*03:01:01 / DQB1*05:01:24*	*DRB1*14:54:01 / DRB1*15:02:01*
CTBD184	*A*24:02:01 / A*24:20:01*	*B*46:01:01 / B*54:01:01*	*C*01:02:01 / C*01:02:01*	*DPB1*05:01:01 / DPB1*05:01:01*	*DQB1*04:02:01 / DQB1*03:03:02*	*DRB1*14:06:01 / DRB1*03:02:01*
CTBD185	*A*24:02:01 / A*24:20:01*	*B*46:01:01 / B*51:01:01*	*C*01:02:01 / C*14:02:01*	*DPB1*14:01:01 / DPB1*13:01:01*	*DQB1*03:03:02 / DQB1*04:02:01*	*DRB1*14:02:01 / DRB1*03:02:01*
CTBD186	*A*33:03:01 / A*11:01:01*	*B*40:02:01 / B*44:03:02*	*C*07:06:01 / C*15:02:01*	*DPB1*13:01:01 / DPB1*05:01:01*	*DQB1*03:02:01 / DQB1*02:02:01*	*DRB1*03:02:01 / DRB1*07:01:01*
CTBD187	*A*02:06:01 / A*11:01:01*	*B*48:01:01 / B*40:01:02*	*C*08:01:01 / C*03:04:01*	*DPB1*02:02:01 / DPB1*05:01:01*	*DQB1*03:03:02 / DQB1*03:01:01*	*DRB1*11:01:01 / DRB1*09:01:02*
CTBD188	*A*33:03:01 / A*11:01:01*	*B*40:01:02 / B*44:03:02*	*C*07:06:01 / C*07:02:01*	*DPB1*04:02:01 / DPB1*02:01:02*	*DQB1*06:01:01 / DQB1*03:02:01*	*DRB1*08:03:02 / DRB1*04:03:01*
CTBD189	*A*24:02:01 / A*11:01:01*	*B*40:01:02 / B*35:01:01*	*C*03:03:01 / C*03:04:01*	*DPB1*03:01:01 / DPB1*16:01:01*	*DQB1*03:02:01 / DQB1*03:01:01*	*DRB1*03:02:01 / DRB1*14:54:01*
CTBD190	*A*11:01:01 / A*02:01:13*	*B*40:02:01 / B*15:02:01*	*C*08:01:01 / C*03:03:01*	*DPB1*14:01:01 / DPB1*05:01:01*	*DQB1*04:02:01 / DQB1*05:02:01*	*DRB1*11:01:01 / DRB1*16:02:01*
CTBD191	*A*11:01:01 / A*24:02:01*	*B*40:06:01 / B*15:02:01*	*C*08:01:01 / C*12:02:02*	*DPB1*21:01 / DPB1*04:01:01*	*DQB1*05:02:01 / DQB1*03:02:01*	*DRB1*14:54:01 / DRB1*04:03:01*
CTBD192	*A*02:03:01 / A*24:02:01*	*B*54:01:01 / B*35:01:01*	*C*01:02:01 / C*03:03:01*	*DPB1*02:02:01 / DPB1*02:01:02*	*DQB1*06:02:01 / DQB1*03:01:01*	*DRB1*11:01:01 / DRB1*14:05:01*
CTBD193	*A*24:02:01 / A*32:01:01*	*B*27:04:01 / B*37:01:01*	*C*04:01:01 / C*03:04:01*	*DPB1*09:01:01 / DPB1*02:01:02*	*DQB1*05:01:01 / DQB1*03:03:02*	*DRB1*10:01:01 / DRB1*09:01:02*
CTBD194	*A*24:03:01 / A*03:01:01*	*B*35:01:01 / B*18:01:01*	*C*04:01:01 / C*07:01:01*	*DPB1*31:01:01 / DPB1*04:01:01*	*DQB1*05:01:01 / DQB1*05:02:01*	*DRB1*13:07:01 / DRB1*01:01:01*
